# Adhesion‐Related Macrophages Regulate Metabolic Homeostasis Through CAV‐1 Dependency

**DOI:** 10.1002/advs.202520936

**Published:** 2026-03-12

**Authors:** Wanyu Hu, Xiyan Liao, Limin Xie, Xiaoxiao Sun, Luodi Jing, Qin Zeng, Hongwei Jiang, Haowei Zhang, Lei Li, Fanqi Wang, Dandan Wang, Ying Mei, Yujin Din, Jianfeng Song, Wufuer Aini, Hui Zhou, Wanqin Xie, Helong Dai, Wei Liu, Joey Liu, Yan Cheng, Feng Liu, Willa A. Hsueh, Tuo Deng

**Affiliations:** ^1^ National Clinical Research Center for Endocrine and Metabolic Diseases Key Laboratory of Diabetes Immunology, Ministry of Education and Department of Metabolism and Endocrinology and Metabolic Syndrome Research Center The Second Xiangya Hospital of Central South University Changsha Hunan China; ^2^ Department of Endocrinology and Metabolism The First Affiliated Hospital of Zhengzhou University Zhengzhou Henan China; ^3^ Endocrine and Metabolic Disease Center First Affiliated Hospital College of Clinical Medicine of Henan University of Science and Technology Luoyang Henan China; ^4^ Department of Orthopedics The First Affiliated Hospital Hengyang Medical School University of South China Hengyang Hunan China; ^5^ Department of Clinical Laboratory Sun Yat‐Sen Memorial Hospital of Sun Yat‐Sen University Guangzhou Guangdong China; ^6^ NHC Key Laboratory of Birth Defect For Research and Prevention Hunan Provincial Maternal and Child Health Care Hospital Changsha Hunan China; ^7^ Department of Kidney Transplantation Center of Organ Transplantation The Second Xiangya Hospital of Central South University Changsha Hunan China; ^8^ Department of Biliopancreatic Surgery and Bariatric Surgery The Second Xiangya Hospital of Central South University Changsha Hunan China; ^9^ Diabetes and Metabolism Research Center Division of Endocrinology Diabetes & Metabolism Department of Internal Medicine Wexner Medical Center of The Ohio State University Columbus Ohio USA; ^10^ Department of Pharmacy The Second Xiangya Hospital of Central South University Changsha Hunan China; ^11^ Hunan Provincial Engineering Research Centre of Translational Medicine and Innovative Drug Changsha China; ^12^ FuRong Laboratory Changsha Hunan China

**Keywords:** adipose tissue, macrophages, CAV‐1, obesity, insulin resistance

## Abstract

The significance of adipose tissue macrophages (ATMs) in regulating adipose tissue function is well‐established. However, our investigation revealed a previously overlooked subpopulation of macrophages adhered to adipocytes, which we term adhesion‐related macrophages (ARMs). We developed an approach to isolate ARMs and compared them with stromal vascular fraction (SVF) macrophages (SMs). Our findings demonstrate that ARMs constitute the predominant expanded subpopulation of ATMs during obesity, ARMs acquire adipocyte mRNA through direct adhesion to adipocytes, thereby enhancing their lipid processing capacity. Notably, ARMs can be characterized by a key functional marker, Caveolin‐1. Genetic ablation of Caveolin‐1 in macrophages significantly diminishes ARM abundance, disrupting their adhesion capacity and lipid content, leading to adipocyte hypertrophy, adipose tissue expansion, and impaired glucose homeostasis. Reintroducing ARMs from lean mice into epididymal white adipose tissue (eWAT) mitigates obesity‐induced insulin resistance. Our study uncovers ARM as a potential therapeutic target for obesity‐induced insulin resistance and opening avenues for identifying similar paradigms in other tissues and diseases.

## Graphical abstract

Adipose tissue harbors a distinct macrophage subpopulation, termed adhesion‐related macrophages (ARMs), which stably adhere to adipocytes. In obesity, ARMs represent the major expanding macrophage subset. They acquire material from adipocytes and rely on Caveolin‐1 for sustained lipid handling. This work identifies intercellular adhesion as a crucial macrophage function in regulating adipose tissue homeostasis and systemic metabolism.

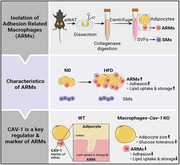

## 1 Introduction

Obesity has become a significant public health concern. Over half of the world's population is currently overweight or obese. Obesity leads to an elevated risk of a series of complications, including type 2 diabetes, fatty liver, hypertension, cardiovascular diseases, and various types of cancer [1]. Obesity‐induced adipose inflammation has been identified as a mediator between obesity and its complications [2]. Macrophages, as the predominant immune cell population in adipose tissue, are pivotal in promoting adipose tissue inflammation [3, 4]. During obesity, they accumulate in adipose tissue, promoting inflammation by upregulating inflammatory factors and interacting with various immunocytes [5, 6].

The nonimmune functions of adipose tissue macrophages (ATMs), such as promoting fibrosis (LY6A^+^ macrophages) [7], stimulating angiogenesis (vascular‐associated macrophages, VAMs) [8], regulating the sympathetic nervous system (sympathetic neuron‐associated macrophages, SAMs) [9], and modulating lipid metabolism (lipid‐associated macrophages, LAMs, and metabolic activation macrophages, MMe macrophges) [10, 11], have become more apparent as our understanding of their heterogeneity has progressed. Among these multifaceted functions, LAMs accumulate during obesity and serve to buffer excessive lipids‐induced lipotoxicity, maintaining systemic homeostasis [10].

Standard ATM isolation procedures have been established to examine the function of ATMs [12]. Adipose tissue was chopped into small pieces and digested into a cellular suspension. Following centrifugation, the uppermost layer of the centrifuge tube is occupied by adipocytes, whereas the stromal vascular fraction (SVF) settles to the bottom. ATMs were isolated from SVF using beads or flow cytometry (FCM). Collagenase is the primary enzyme for adipose tissue digestion [10, 13, 14] and ATMs isolation due to its high cellular yield and ability to preserve cell surface proteins [15, 16]. After confirming sufficient collagenase digestion, we observed the presence of macrophage contaminants within the adipocyte layer during the isolation of adipocytes from both mice and humans [17]. To eliminate macrophage contamination and obtain pure adipocytes, we incubated crudely isolated adipocytes with anti‐CD45 magnetic beads [17]. We found that the macrophages within the adipocyte layer adhered to individual adipocytes, as also observed by Ebke et al. [18] These adhesion‐related macrophages (ARMs) interact closely with adipocytes and potentially play a crucial role in the maintenance of adipose tissue homeostasis. However, they have been overlooked in previous studies of ATM, leaving their characteristics and functions unknown.

In this study, we developed a method to isolate ARMs from white adipose tissues based on their adherence to adipocytes. Our findings indicate that ARMs constitute a distinct subset of ATMs that significantly expand during obesity and comprise the majority of ATMs in obese mice. ARMs acquire adipocyte mRNA through direct adhesion to adipocytes, thereby enhancing their lipid processing capacity. We identified Caveolin‐1 (CAV‐1) as a surface marker for ARMs and a key regulator of their adhesion and lipid processing properties. CAV‐1 deletion markedly reduces ARMs, leading to expanded adipose tissue and systemic glucose intolerance during obesity. Supplementation of ARMs improves glucose metabolism in obese mice. These findings suggest that ARMs are a distinct macrophage subpopulation that maintain adipose tissue metabolic homeostasis by acquiring adipocyte mRNA through adhesion.

## 2 Results

### 2.1 Obesity Elicits an Accumulation of ARMs

**FIGURE 1:**
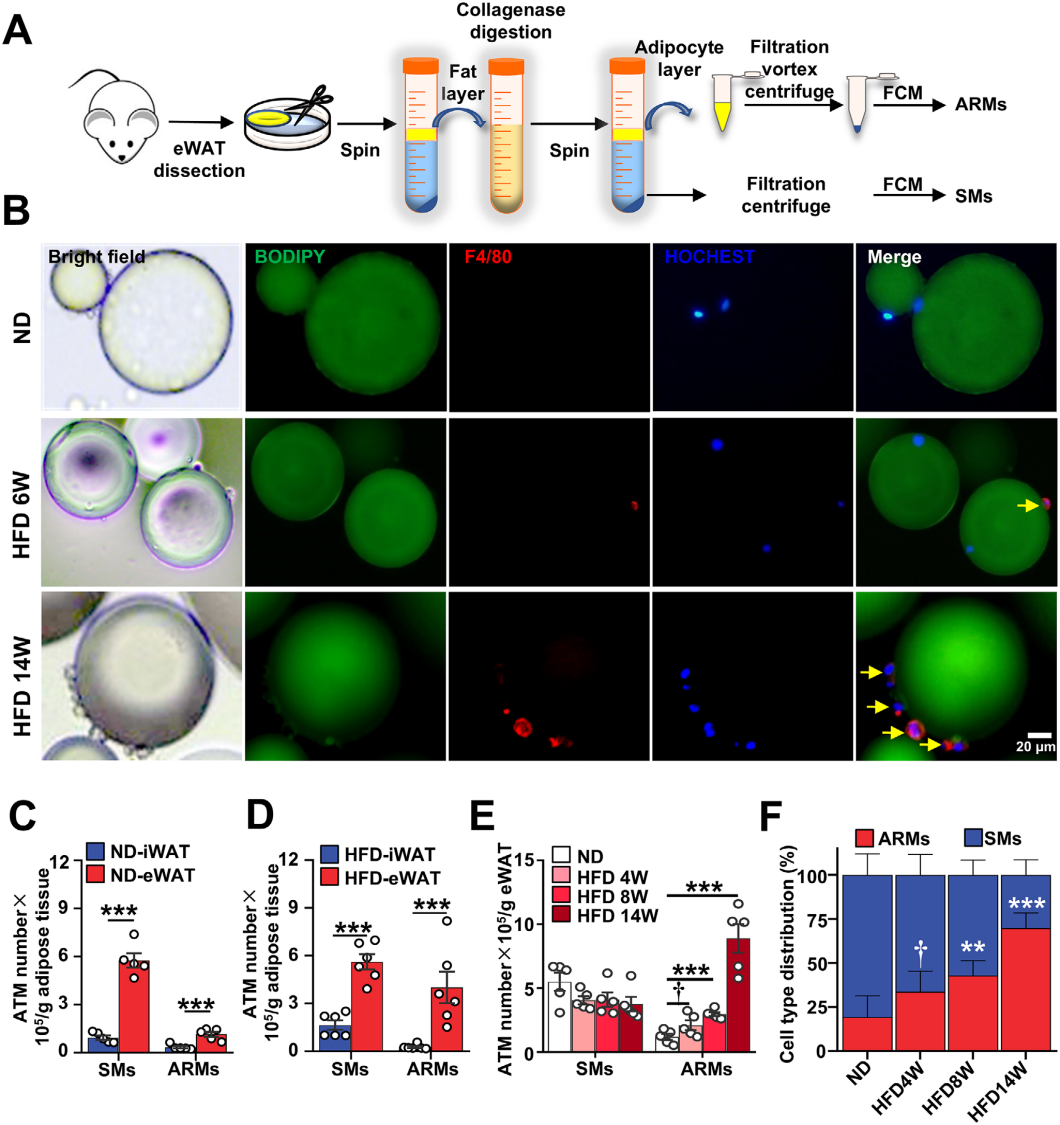
Obesity elicits an accumulation of adhesion‐related macrophages (ARMs). (A) Schematic illustration depicting the isolation process of ARMs and stromal vascular fraction (SVF) macrophages (SMs) from eWAT. The eWAT underwent mincing, washing, and collagenase digestion. Subsequent centrifugation separated adipocytes from the SVF. Adipocytes were filtrated, vortexed, and centrifuged to collect the cellular pellet, from which ARMs were isolated using flow cytometry (FCM) or bead purification. Similarly, SVF were filtered to retrieve SMs by FCM. (B) Microscopy image of ARMs adhered to adipocytes in eWAT of both normal diet (ND) and high‐fat diet (HFD) mice. Scale bar, 20 µm. (C,D) FCM analysis of ARMs and SMs abundance in iWAT and eWAT from (C) age‐matched ND mice and (D) HFD 8‐week mice. *n* ≥ 5 per group. (E,F) FCM analysis of (E) ARMs and SMs abundance in eWAT from mice with prolonged exposure to HFD and (F) cell type distribution within ATM compartment. *n* ≥ 5 per group. Data are expressed as means ± SEM. †*p* < 0.1, ** *p* < 0.01, ***p < 0.001.

Little is currently known about ARM due to the lack of reliable methods to isolate ARMs. To investigate this subpopulation of macrophages, we developed a method for isolating ARM. Adipose tissue sample was minced into fragments of approximately 8 mm^3^ in phosphate‐buffered saline (PBS). Tissue suspensions were centrifuged and treated with collagenase. The subsequent centrifugation effectively separated floating adipocytes from the SVF pellet (Figure 1A). The adipocyte layer is composed of adipocytes and cells that are attached to adipocytes, including ARMs. The observation of live adipocytes surrounding ARMs in epididymal white adipose tissue (eWAT) of mice fed a high‐fat diet (HFD) via fluorescent microscopy suggests that ARMs were preserved in the adipocyte layer and increase gradually with prolonged exposure to HFD (Figure 1B; Figure S1B). Considering the vulnerability of adipocytes to mechanical stress and drastic temperature changes [19, 20], we utilized vortex oscillation with 1 mm beads to disrupt adipocytes, followed by low‐temperature centrifugation to obtain a cell pellet containing ARMs. By using FCM sorting, macrophages were obtained from these cells and the SVFs, exhibiting minimal contamination from other cellular components; these macrophages were designated as ARMs and SVF macrophages (SMs), respectively (Figure 1A; Figure S1A). Type I, II, and IV collagenases were compared under commonly used conditions, testing two incubation times (20 and 30 min) and concentrations (1 and 2 mg/mL). ARM yield and cell viability were comparable across all conditions. The commonly used adipose tissue protocol of 1 mg/mL type II collagenase digestion for 30 min is suitable for ARM isolation and was retained for consistency in subsequent experiments (Figure S1C,D). 0.25% trypsin digestion (2 min) dissociates adipocyte–ARM interactions, providing an alternative to vortexing under less stringent surface protein requirements (Figure S1E,F).

Next, we examined the distribution of ARMs in inguinal WAT (iWAT) and eWAT from normal diet (ND)‐ and HFD‐fed mice. Flow cytometric analysis revealed that eWAT exhibited a much higher abundance of ARMs and SMs than iWAT under both ND and HFD conditions (Figure 1C,D). Additionally, obesity led to an increase of ARMs in eWAT, but not in iWAT (Figure 1C,D). To elucidate the changes in the abundance of ARM during the progression of obesity, we utilized FCM to analyze the dynamics of ARMs in eWAT from mice subjected to varying durations of HFD. Consistent with previous reports [21, 22], we observed a significant increase in the total number of SMs in the fat pad during HFD (Figure S1G,H). After weight normalization, the number of SMs remained stable during the HFD process. However, the numbers and proportions of ARMs in ATMs increased as obesity progressed (Figure 1E,F). By week 14 of HFD, ARMs constituted over 70% of ATMs in eWAT (Figure 1F), indicating that ARMs represent the predominant ATM population associated with obesity.

As collagenase is commonly used to isolate macrophages from various tissues, we next sought evidence for the presence of ARMs beyond adipose tissue. As depicted in Figure S2A, hepatocyte suspension was obtained after in vivo collagenase digestion, followed by washing with PBS 3 times. Immunofluorescence staining revealed the adhesion of macrophages to hepatocytes (Figure S2B). Subsequent trypsin digestion disrupted their connection. The cells in the wash solution and trypsin‐digested liver cells were separately subjected to percoll separation, resulting in a single nuclear cell layer for FCM analysis, representing either hepatic macrophages (HMs) or ARMs, respectively (Figure S2A). Notably, the ARMs constituting over 10% of total liver macrophages (Figure S2C). Furthermore, we explored the presence of ARMs in tumors. Mouse subcutaneous MC38 tumors were collected and subsequently dissociated by shredding and collagenase digestion. The resulting cells were separated by percoll, yielding an upper layer of tissue cells and an intermediate layer of single nuclear cells (Figure S2D). Immunofluorescence staining revealed ARMs adhering to CD45 ***^−^*** cells, predominantly tumor cells, in the upper layer of tissue cells (Figure S2E). Subsequent trypsin digestion dissociated ARMs from tumor cells. ARMs within the tissue layer and conventional tumor‐associated macrophages (TAMs) in single nuclear cells were analyzed by FCM (Figure S2D). ARMs accounted for approximately 40% of the total tumor macrophages (Figure S2F). These findings establish ARM as a conserved macrophage subpopulation in mouse adipose tissue, liver, and tumors.

### 2.2 SM to ARM Transformation and In Situ Proliferation Drive ARM Accumulation in Obesity

To elucidate the mechanism underlying the accumulation of ARMs in eWAT during obesity, we performed FCM analyses to determine the proliferation of ARMs and SMs from mice under ND and HFD by Ki67 staining (Figure S3A) and EdU incorporation (Figure S3B). HFD feeding promoted proliferation in both ARMs and SMs (Figure S3A,B). Notably, ARMs exhibited approximately twofold increase in proliferation rate compared to SMs in both ND and HFD conditions (Figure S3A,B). This suggests that an increased degree of proliferation contributes to the obesity‐induced accumulation of ARMs.

Next, to investigate the contribution of monocyte infiltration to the accumulation of ARMs under HFD conditions, we developed an irradiation‐bone marrow transplantation chimera mouse model. The lower abdomens of CD45.2 mice were shielded with lead to protect the resident macrophages in eWAT. These CD45.2 mice were exposed to lethal irradiation and subsequently were transplanted with bone marrow from CD45.1 mice. After 6 weeks of chimerism, recipient CD45.2 mice were subjected to 8 or 14 weeks of HFD, and the chimerism rate was evaluated by FCM analysis on CD45.1 cells (Figure S3C). Chimerism was successfully established, with approximately 40% of circulating monocytes being donor derived (CD45.1^+^), and no significant difference observed between ND and HFD groups (Figure S3D). At 8 weeks of HFD feeding, the proportion of CD45.1^+^ cells in both ARMs and SMs remained comparable to that in ND controls. In contrast, 14 weeks of HFD feeding resulted in a significant increase in donor‐derived cells within both subsets (Figure S3E). These findings suggest that monocyte infiltration plays a negligible role in ATM accumulation during early obesity but contributes modestly under prolonged HFD exposure, consistent with previous reports [23]. Notably, under both dietary conditions, ARMs harbored a lower proportion of donor‐derived (CD45.1^+^) cells than SMs (Figure S3D), indicating that the elevated ARM/SM ratio observed in obesity is not attributable to monocyte recruitment.

Finally, to examine whether HFD could induce the shift of SMs into ARMs, we performed SMs transfer experiments by injecting CFSE‐labeled SMs from ND mice into eWAT of ND or HFD mice and evaluated the proportion of CFSE‐labeled macrophages in ARMs and SMs after 2 days of injection (Figure S3F). In the eWAT of ND mice, only 20% of CFSE‐labeled SMs transform into ARMs. However, under HFD conditions, this proportion significantly rises to nearly 50% (Figure S3G). This result suggests a higher propensity for SMs cells to transform into ARM under HFD conditions compared to ND conditions. Notably, SMs that converted to ARMs exhibited higher CAV‐1 expression (Figure S3H), while CAV‐1 knockout reduced SM‐to‐ARM conversion (Figure S3I). Together, the accumulation of ARMs in eWAT induced by HFD is mainly attributed to a combination of increased proliferation and transformation from SMs.

### 2.3 ARMs Exhibit a Gene Landscape for Adhesion and Lipid Accumulation

**FIGURE 2:**
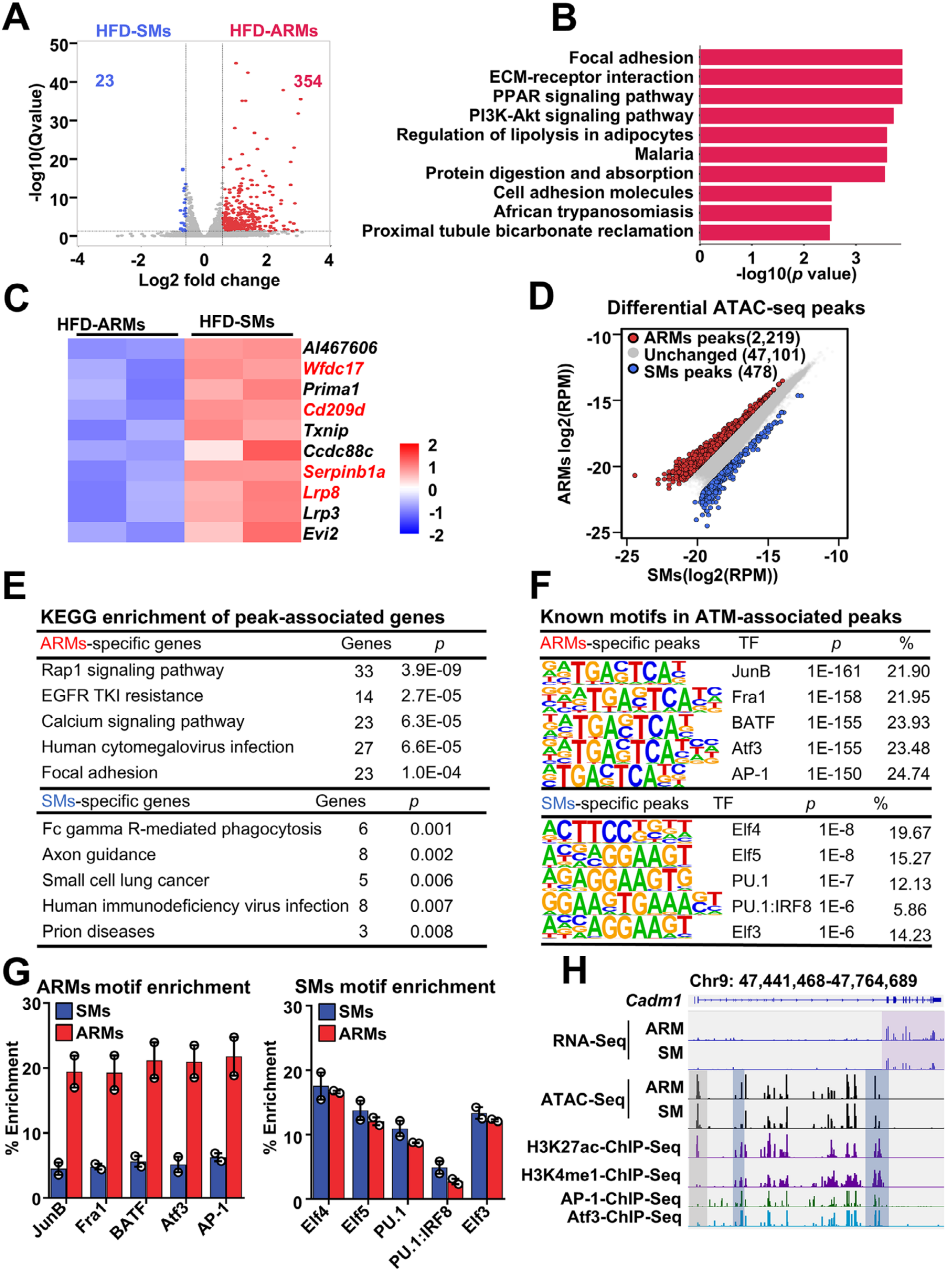
ARMs exhibit a gene landscape for adhesion and lipid accumulation. (A) Volcano plot illustrating the differentially expressed genes between FACS‐sorted ARMs and SMs from mice exposed to an HFD for 8 weeks (fold change > 1.5, false discovery rate < 0.05, *n* = 2 per group, with 6 mice pooled per sample). (B) KEGG pathway analysis of ARMs upregulated gene, depicting the ten most significant pathways. (C) Heat map of the top 10 downregulated genes between SMs and ARMs. (D) Differential analysis of ATAC‐seq peaks from ARMs and SMs of mice on an HFD for 8 weeks (fold change > 2, false discovery rate < 0.05, *n* = 2 per group, with 6 mice pooled per sample). (E) KEGG pathway analysis for genes associated with ATAC‐seq peaks in ARMs and SMs. Most significant signaling pathway with gene number and adjusted *p* value are shown. (F) Homer known motifs present in peaks associated with ARMs and SMs (consensus motif, transcription factor (TF), *p* value, and percentage of targets shown). (G) Percent enrichment of AP‐1 family motifs and ETS family motifs within ARM‐ or SM‐specific peaks. (H) Representative RNA‐seq, ATAC‐seq, and ChIP‐seq browser tracks displaying ARM‐specific gene loci. Legacy ChIP‐seq tracks for AP‐1, ATF3, H3K27ac, and H3K4me1 in BMDM are displayed.

To determine the transcription signature of ARMs, RNA‐seq analysis was conducted on ARMs and SMs that were sort‐purified from the eWAT of mice subjected to ND or an 8‐week HFD. This analysis identified 258 ARM‐specific genes and 8 SM‐specific genes in ND mice (Figure S4A), as well as 354 ARM‐specific genes and 23 SM‐specific genes in HFD mice (Figure 2A). Unbiased clustering and Kyoto Encyclopedia of Genes and Genomes (KEGG) analysis indicated that genes predominantly expressed in the ARMs of HFD mice were enriched for pathways associated with cell adhesion and lipid metabolism (Figure 2B). SM‐specific genes were related to phagocytosis and endocytosis (*Lpr8* and *Cd209d*), and bacteria clearance (*Serpinb1a*, *Wfdc17*) (Figure 2C). Notably, KEGG pathways enriched in ARMs from ND mice closely resemble those in HFD mice, as several adhesion‐related pathways enriched in ND‐ARMs are also found in HFD‐ARMs (Figure S4B). These results indicate that ARMs and SMs activate distinct transcriptional pathways, potentially mediated by cell type‐specific regulatory elements, particularly within ARMs.

To identify the cell type‐specific chromatin regulatory elements and transcription factors (TFs) driving the functionally divergent transcriptomes of ARMs or SMs, we performed an Assay for Transposase Accessible Chromatin with sequencing (ATAC‐seq). Differential peak analysis identified 2219 ARM‐specific and 478 SM‐specific ATAC‐seq peaks, indicating distinct chromatin accessibility patterns between these cell subpopulations (Figure 2D). Analyzing the cell type‐specific genes linked to their respective peaks through KEGG analysis validated the cell adhesion pathways (Rap1 signaling and focal adhesion pathways) and FcγR‐mediated phagocytosis gene programs of ARMs and SMs (Figure 2E). To elucidate the TFs responsible for driving ARM and SM‐specific gene programs, we conducted a known motif search within cell type‐specific peaks. The ARM‐specific peaks were enriched for AP‐1 family TFs, including JunB, Fra1, BATF, Atf3, and AP‐1, whereas SM‐specific peaks were dominated by ETS family factors (Elf4, Elf5, PU.1, PU.1–IRF8, and Elf3) with only a marginal increase (Figure 2F,G). Western blotting showed increased AP‐1 expression in HFD ARMs, while PU.1 protein levels were similar between ARMs and SMs (Figure S5A). An integrated analysis of macrophage cistromes from publicly available datasets revealed that the binding of AP‐1 and ATF3 were enriched at ARM‐specific peaks, indicating a high degree of correlation between TF binding sites and ARM‐specific enhancers to maintain adhesion‐related gene transcription (for example, *Cadm1*; Figure 2H).

To determine the proteomic signature of ARMs, proteomic analysis was performed on ARMs and SMs sort‐purified from eWAT of mice fed ND or an 8‐week HFD. In ND mice, 67 ARM‐specific and 34 SM‐specific proteins were identified (Figure S6A), whereas in HFD mice, 162 ARM‐specific and 68 SM‐specific proteins were detected (Figure S6C). KEGG analysis indicated that ARM‐enriched proteins were predominantly associated with cell adhesion and lipid‐processing pathways (Figure S6B,D). Compared to ND mice, both ARMs and SMs under HFD exhibit a low‐grade proinflammatory state (Figure S7). In summary, the distinctive transcriptomes of ARMs, which are partly shaped by unique chromatin landscapes activated by specific combinations of TFs, indicate that ARMs have an increased capacity for cell adhesion and lipid processing.

### 2.4 ARMs Exhibit Robust Adhesive Properties

**FIGURE 3:**
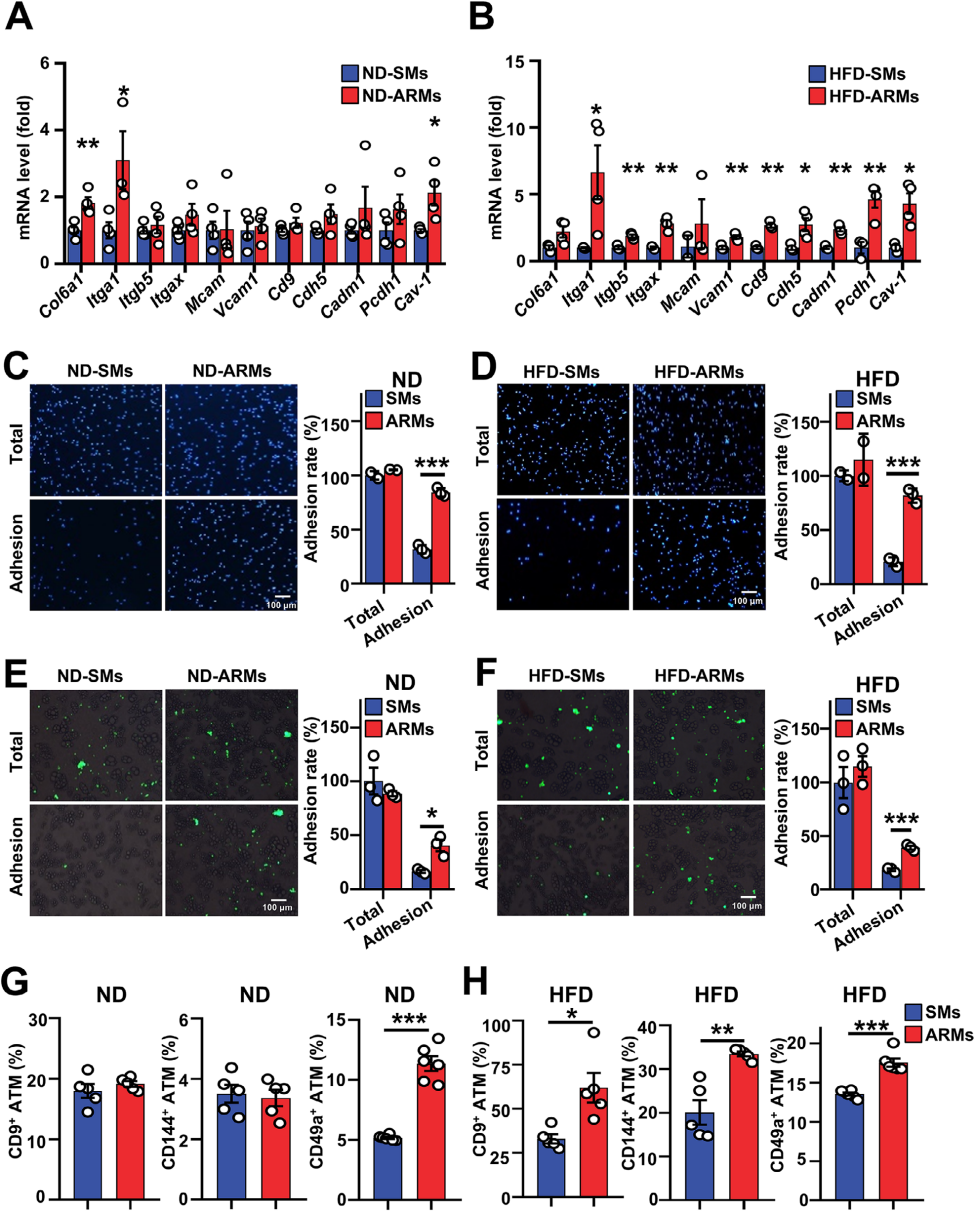
ARMs exhibit robust adhesive properties. (A,B) Gene expression profiles of ARMs and SMs isolated from (A) ND mice and (B) HFD 8 W mice (*n* =3–5 per group, with 3 mice pooled per sample). (C,D) Culture‐dish attachment assay. ARMs and SMs from (C) ND mice and (D) HFD 8 W mice were beads‐purified and placed on the matrigel‐coated cell culture‐dish. After 30 min of incubation, the cells were washed and fixed. Cell nuclei were stained with blue (DAPI). The remaining adherent cells on the dish were counted (*n* = 3, Scale bar, 100 µm). (E,F) Adipocyte attachment assay. ARMs and SMs from (E) ND mice and (F) HFD 8 W mice were beads‐purified, CFSE‐labeled and placed on the adipocytes differentiated from Adipose‐derived stem cells (ASCs). After 30 min of incubation, the cells were washed and fixed. Cells are shown in green (CFSE). The remaining adherent cells on the adipocytes were counted (*n* = 3, Scale bar, 100 µm). (G,H) FCM analysis of adhesion molecules in ARMs and SMs from (G) ND and (H) HFD 8 W mice (*n* =5–6 per group). Data are expressed as means ± SEM. *p < 0.05, **p < 0.01, ***p < 0.001.

ARMs are strongly attached to adipocytes and cannot be disassociated from adipocytes by collagenase digestion. The RNA‐seq data indicates heightened expression patterns of adhesion‐related genes in ARMs (Figure 2B). To confirm these RNA‐seq data, we conducted reverse transcriptase‐ polymerase chain reaction (RT‐PCR) analysis to compare the gene expression of adhesion‐related genes in both ARMs and SMs. The mRNA levels of cell‐matrix adhesion molecules (*Col6a1*, *Itga1*, *Itgb5*, and *Itgax*) and cell–cell adhesion molecules (*Itga1*, *Itgb5, Itgax, Mcam*, *Vcam*, *Cd9*, *Cdh5*, *Cadm1*, *Pcdh1*, and *Cav1*) were found to be significantly higher in ARMs compared to SMs (Figure 3A,B). To verify the differences in adhesion capacity between ARMs and SMs, we initially examined the attachment of ARMs and SMs on matrigel‐coated cell culture dishes. ARMs from either ND‐ or HFD‐fed mice displayed a higher adhesion rate on the cell culture dish than SMs (Figure 3C,D). Next, we performed a macrophage‐adipocyte adhesion assay. Consistently, ARMs from both ND‐fed and HFD‐fed mice exhibited higher adhesion rates than SMs on the surface of adipocytes (Figure 3E,F). As the majority of adhesion molecules are expressed on the cell membrane surface, we utilized FCM surface staining to measure the expression of cell adhesion molecules, including CD144 (CDH5), CD9, and CD49a (ITGA1), on the cell membranes of ARMs and SMs. In obese mice, ARMs exhibited heightened levels of CD144, CD9, and CD49a, while CD49a increased in lean mice (Figure 3G,H). Pretreatment with BIO1211 did not reduce the adhesion rate between ARMs and adipocytes (Figure S8A), indicating that α4 integrin is unlikely to mediate these interactions in our experimental system, although it has been reported to participate in adipocyte–macrophage adhesion [24]. Together, these data indicate that ARMs have enhanced adhesion capability.

### 2.5 ARMs Possess a Robust Capacity for Lipid Accumulation

**FIGURE 4:**
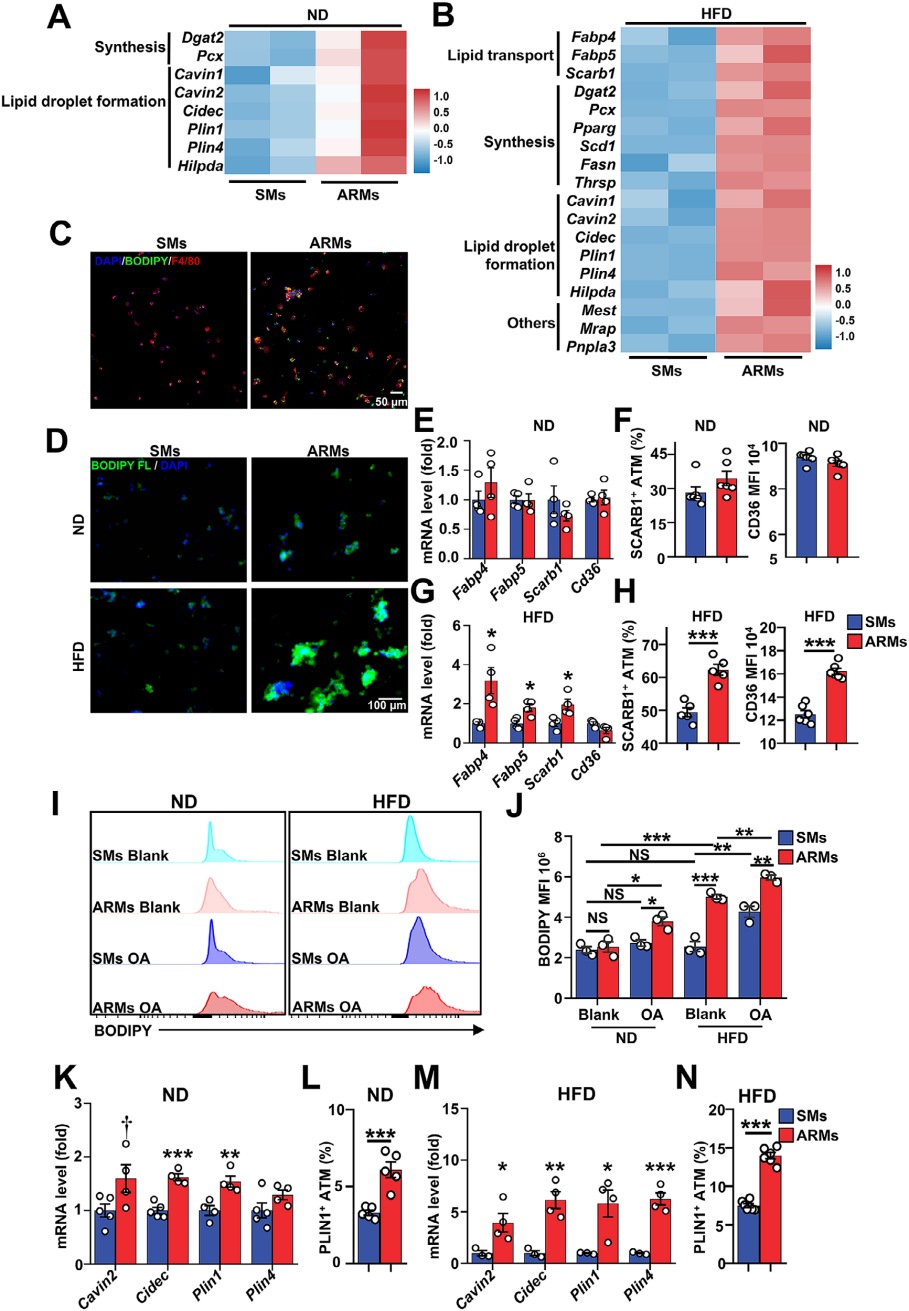
ARMs possess a robust capacity for lipid accumulation. (A,B) Heatmap displaying the expression of lipid processing genes in (A) ND mice and (B) HFD mice between SMs and ARMs. (C) Representative immunofluorescence images of BODIPY (green), F4/80 (red), and nuclei (blue) in ARMs and SMs isolated from HFD 8 W mice. Scale bar, 20 µm. (D) Fatty acid trafficking in ARMs and SMs isolated from ND or HFD 8 W mice loaded with BODIPY FL. C12 for 30 min. Scale bar, 100 µm. (E) Gene expression profiles of lipid transport in ARMs and SMs isolated from ND mice (*n* = 4 per group, with 3 mice pooled per sample). (F) FCM analysis of SCARB1 and CD36 in ARMs and SMs from ND mice (*n* = 6 per group). (G) Gene expression profiles of lipid transport in ARMs and SMs isolated from HFD 8 W mice (*n* =4 per group, with 3 mice pooled per sample). (H) FCM analysis of SCARB1 and CD36 in ARMs and SMs from HFD 8 W mice (*n* = 5–6 per group). (I) FCM analysis of BODIPY staining in ARMs and SMs from ND and HFD 6 W mice after lipid‐loaded with oleate acid (OA) for 4 h or bovine serum albumin (BSA) (Blank). (J) Quantification of BODIPY mean fluorescence intensity (MFI) levels (*n* = 3). (K) Gene expression profiles of lipid droplets formation in ARMs and SMs isolated from ND mice (*n* = 4–5 per group, with 3 mice pooled per sample). (L) FCM analysis of PLIN1 in ARMs and SMs from ND mice (*n* = 5 per group). (M) Gene expression profiles of lipid droplets formation in ARMs and SMs isolated from HFD 8 W mice (*n* = 4–5 per group, with 3 mice pooled per sample). (N) FCM analysis of PLIN1 in ARMs and SMs from HFD 8 W mice (*n* = 6 per group). Data are expressed as means ± SEM. †*p* < 0.1, *p < 0.05, **p < 0.01, ***p < 0.001, NS = not significant.

Transcriptome analysis revealed a significant enrichment of genes associated with lipid metabolism in ARMs. In ND mice, ARMs exhibited heightened expression of genes related to various aspects of lipid metabolism, including lipid synthesis (*Dgat2*, *Pcx*), and lipid droplet formation (*Cavin1*, *Cavin2*, *Cidec*, *Hilpda*, *Plin1*, and *Plin4*) (Figure 4A). In addition to the upregulated gene profile observed under ND conditions, further lipid processing‐related characteristics were exhibited by ARMs in the context of HFD, including fatty acid transport (*Fabp4*, *Fabp5*, *Scarb1*), de novo lipid synthesis (*Fasn*, *Thrsp*, *Scd1*), and lipogenesis (*Pparg*) (Figure 4B). Gene signature analysis suggests that ARMs regulate lipid metabolism in adipose tissue, promoting lipid synthesis and stable neutral lipid storage, especially under HFD conditions. Indeed, larger and more numerous neutral lipid droplets are present in the ARMs of HFD‐fed mice compared to the SMs of the same group of mice (Figure 4C).

To determine whether the augmented lipid storage in ARMs results from increased lipid uptake, we quantified fatty acid uptake 25 min after the addition of BODIPY FL. C12. Fluorescence microscopy revealed that there was no significant difference in fluorescence intensity between ARMs and SMs from ND mice. However, under HFD conditions, ARMs exhibited markedly higher fluorescence intensity compared to SMs (Figure 4D). Moreover, under ND conditions, there were no statistically significant disparities detected in the expression of genes linked to lipid transport, such as *Fabp4*, *Fabp5*, *Scarb1*, and *cd36* between ARMs and SMs (Figure 4E). Consistently, since SCARB1 and CD36 facilitates fatty acid uptake [25], we assessed the protein expression of SCARB1 and CD36 in ATMs. The ratio of SCARB1^+^ and CD36^+^ showed no significant variation in SMs and ARMs in ND mice (Figure 4F). Under HFD conditions, ARMs showed significantly increased expression of *Fabp4*, *Fabp5*, and *Scarb1* compared with SMs (Figure 4G). Additionally, ARMs exhibited a higher ratio of SCARB1^+^ and CD36^+^ compared to SMs (Figure 4H). These results indicate that ARMs have an enhanced capacity for lipid uptake under HFD conditions.

Next, to test whether ARMs exhibit superior efficiency in converting fatty acids into neutral lipids for storage, we conducted FCM analysis of BODIPY staining to measure intracellular neutral lipid accumulation in ARMs and SMs incubated with oleic acid (OA). Under ND conditions, ARMs and SMs displayed comparable baseline levels of intracellular lipids (Figure 4I,J). However, upon OA treatment, lipid content significantly increased in ARM but remained unchanged in SMs (Figure 4I,J), suggesting that ARMs exhibit a greater capacity to convert exogenous fatty acids into neutral lipids. Under HFD conditions, ARMs exhibited markedly higher baseline intracellular lipid levels than SMs (Figure 4I,J). Moreover, compared to ND‐fed mice, ARMs—but not SMs—showed a significant increase in baseline lipid content, indicating that ARMs may preferentially function as lipid buffers in the obese state. OA treatment further increased neutral lipid accumulation in both subsets under HFD, with ARMs again displaying higher levels than SMs (Figure 4I,J). Consistent with these findings, ARMs expressed higher levels of genes involved in lipid droplet formation, including *Cavin2*, *Cidec*, *Plin1*, and *Plin4* (Figure 4K,M), and exhibited a higher ratio of lipid droplet protein PLIN1^+^ compared to SMs under both ND and HFD conditions (Figure 4L,N). To assess whether lipid accumulation in ARMs reflects reduced fatty acid oxidation, we examined fatty acid beta‐oxidation (FAO)‐related gene expression (Figure S9A,B) and directly measured FAO activity (Figure S9C,D) in ARMs and SMs under ND and HFD, with no significant differences observed. Together, these data indicate that ARMs have enhanced lipid uptake and storage capability.

### 2.6 ARMs Have Lower Engulfment Capacity Under HFD Conditions

Phagocytosis is an essential macrophage function that takes place under a wide range of physiological and pathological conditions [26, 27]. Subsequently, we examine whether ARMs exhibit an elevated ability to uptake substances other than FFA. The phagocytosis of dextran and *Escherichia coli* by SMs and ARMs isolated from eWAT of both ND and HFD mice was compared dextran and *E. coli* phagocytosis did not differ between ARMs and SMs derived from ND mice (Figure S10A,B). However, under HFD conditions, ARMs exhibited diminished phagocytic capacity compared to SMs (Figure S10A,B). To assess the in vivo phagocytosis capacity of ARMs and SMs, we intravenously injected ND‐ or HFD‐fed mice with FITC‐labeled dextran. After a 30 min interval, we performed FCM analysis to evaluate the proportion of dextran^+^ macrophages in eWAT (Figure S10C). ARMs exhibited a lower ratio of dextran^+^ compared to SMs under HFD conditions, with no significant difference under ND conditions (Figure S10D), suggesting a compromised phagocytic capacity in ARMs. Furthermore, ARMs express much lower levels of phagocytosis‐related genes (*Cd209d* and *Wfdc17*) than SMs under HFD conditions but not under ND conditions (Figure S10E,F). Overall, these findings indicate that ARMs do not outperform SMs in engulfing substances such as dextran and bacteria under ND conditions, and their performance may even be inferior under HFD conditions.

### 2.7 CAV‐1 Expression Characterizes ARMs

**FIGURE 5:**
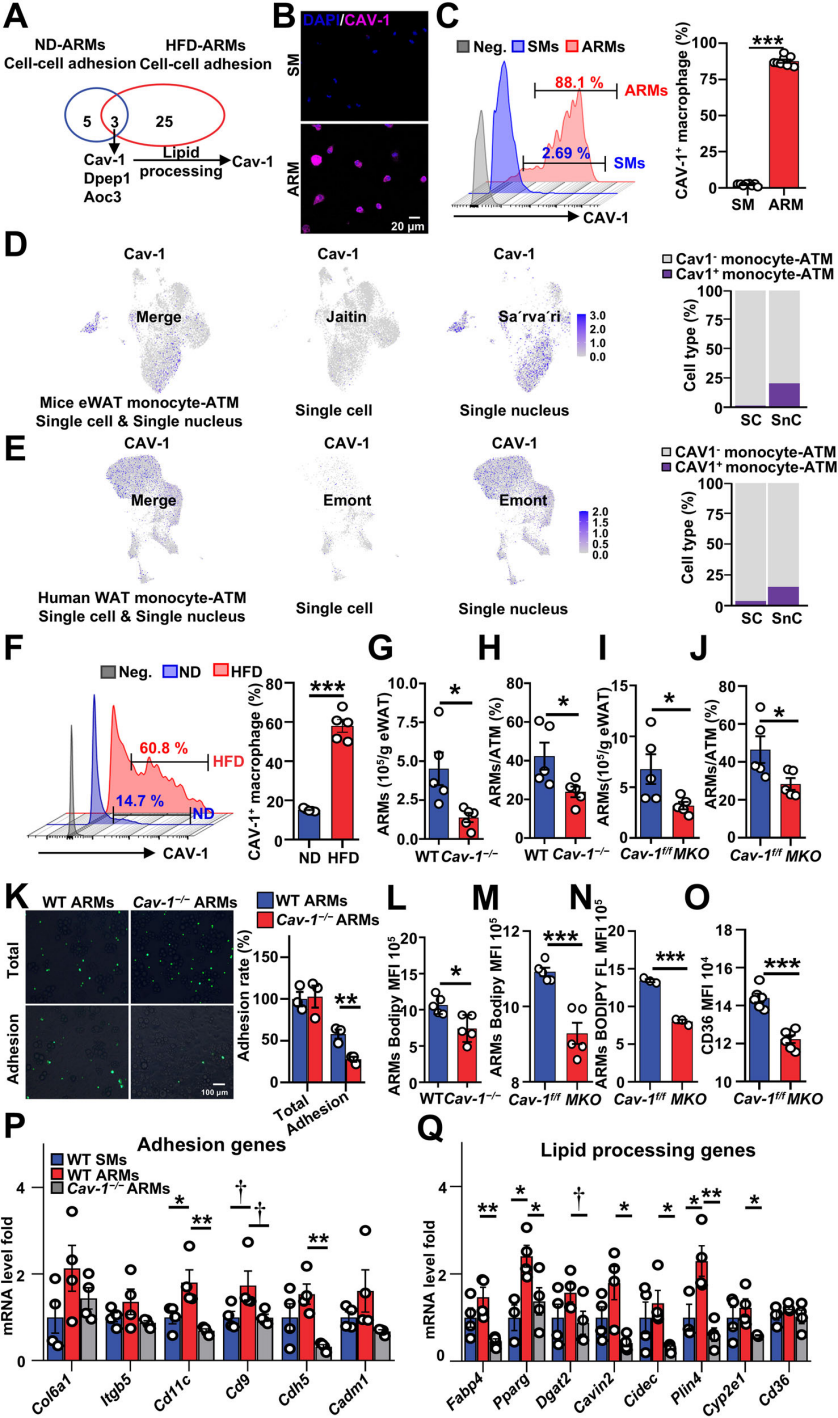
CAV‐1 expression characterizes ARMs. (A) Venn diagram depicting the overlap of cell–cell adhesion genes in ARMs from ND and HFD mice. (B) Representative immunofluorescence images of CAV‐1 (green) and nuclei (blue) in ARMs and SMs isolated from HFD 8 W mice. Scale bar, 50 µm. (C) FCM analysis of CAV‐1^+^ macrophages in ARMs and SMs from HFD 6 W mice (*n* = 8, Neg., ATM FMO). (D) Expression of Cav‐1 in the monocyte‐macrophage compartment in single‐cell & single‐nucleus RNA sequencing of eWAT from mice. (E) Expression of CAV‐1 in the monocyte‐macrophage compartment in single‐cell & single‐nucleus RNA sequencing of WAT from human. (F) FCM analysis of CAV‐1^+^ ATMs in ND and HFD 8 W mice (*n* = 5). (G,H) FCM analysis of ARMs abundance (G), and ARMs distribution (H) within ATM compartment in eWAT from WT and *Cav‐1^−/−^* mice after 10 W HFD (*n* = 5 per group). (I,J) FCM analysis of ARMs abundance (I), and ARMs distribution (J) within ATM compartment in eWAT from *Cav‐1^f/f^* and *MKO* mice after 10 W HFD (*n* = 5 per group). (K) Adipocyte attachment assay. ARMs and SMs from WT and *Cav‐1^−/−^* mice after 10 W HFD were beads‐purified, CFSE‐labeled and placed on the adipocytes differentiated from ASCs. After 30 min of incubation, the cells were washed and fixed. Cells are shown in green (CFSE). The remaining adherent cells on the adipocytes were counted (*n* = 3, Scale bar, 100 µm). (L,M) FCM analysis of BODIPY staining in ARMs from eWAT of (L) WT and *Cav‐1^−/−^* mice after 10 W HFD, and (M) *Cav‐1^f/f^* and *MKO* mice after 10 W HFD (*n* = 5 per group). (N) Fatty acid trafficking in ARMs isolated from HFD *Cav‐1^f/f^* and *MKO* mice loaded with BODIPY FL C12 for 30 min (*n* = 3). (O) FCM analysis of CD36 in ARMs of HFD *Cav‐1^f/f^* and *MKO* mice (*n* = 6 per group). (P,Q) Gene expression profiles of (N) adhesion and (O) lipid processing in ARMs and SMs isolated from WT and *Cav‐1^−/−^* mice after 10 W of HFD (*n* = 3–4 per group, 2 mice pooled to a sample). Data are expressed as means ± SEM. †*p* < 0.1, *p < 0.05, **p < 0.01, ***p < 0.001.

Next, we aim to identify the key regulator of ARM function. Collagens are the major component of extracellular matrix (ECM) [28], and the breakdown of the ECM network by collagenase can disrupt the cell‐matrix interactions while preserving cell–cell adhesion [29, 30, 31]. Since the interaction between ARMs and adipocytes is resistant to collagenase digestion, immunofluorescence further indicates that ARMs directly adhere to adipocytes (Figure 1B). We identified the upregulated genes related to cell–cell adhesion in ARMs under both ND (Figure S11A) and HFD conditions (Figure S11B) through analysis of our RNA‐seq data. The common genes shared between the ND and HFD groups include Cav‐1, Dpep1, and Aoc3. Among them, only CAV‐1 is implicated in both cell adhesion [32, 33, 34, 35] and lipid handling [36, 37, 38]. (Figure 5A) In addition, CAV‐1 is a cell membrane protein and its role as a immunocyte marker remains unexplored [39]. Therefore, we speculate that CAV‐1 is a cell surface marker for ARMs and a key regulator that controls ARM function.

To validate CAV‐1 as an ARM‐specific marker, immunofluorescence and FCM analyses were performed. CAV‐1 is highly expressed in ARMs (Figure 5B,C), with more than 85% of ARMs, while less than 3% of SMs, were found to be CAV‐1^+^ (Figure 5C). We also examined CAV‐1 expression in other immune cells within adipose tissue, including T cells, B cells, and DCs, and found minimal CAV‐1 expression in these cells (Figure S11D). We also reanalyzed published single‐cell and single‐nucleus RNA sequencing datasets from mouse eWAT and human white adipose tissues [10, 40, 41]. Single‐cell RNA sequencing of adipose tissue typically involves collagenase digestion to isolate SVF cells, followed by sequencing. In contrast, single‐nucleus sequencing bypasses tissue dissociation and directly isolates nuclei, thereby preserving the transcriptomic information of all cell types, including those embedded in the adipocyte layer. Theoretically, ARMs, being tightly associated with the adipocyte compartment, would be preferentially captured by single‐nucleus rather than single‐cell sequencing. Indeed, our reanalysis data revealed that CAV‐1^+^ macrophages were readily detectable in single‐nucleus datasets but nearly absent from single‐cell datasets (Figure 5D,E). These findings suggest that CAV‐1^+^ macrophages are largely excluded from the SVF due to their adhesion to adipocytes. In addition, FCM analysis revealed a dramatic increase in the ratio of CAV‐1^+^ ATMs in HFD‐fed obese mice (Figure 5F), mirroring the obesity‐induced accumulation of ARMs. Immunofluorescence analysis also showed obesity‐induced accumulation of CAV‐1^+^ macrophages within the visceral adipose tissue in both mice (Figure S11F) and humans (Figure S11G). CAV‐1 expression in ARMs was higher in HFD‐fed mice than in ND‐fed mice (Figure S11H). Together, CAV‐1 serves as a specific and reliable marker of ARMs.

To determine the essential role of *Cav‐1* in regulating ARM function, we analyzed the ATM compartment in eWAT from *Cav‐1*‐deficient and wild‐type (WT) mice. Obesity‐induced ARM accumulation was significantly reduced in *Cav‐1^−/−^* mice (Figure 5G,H). To further confirm the impact of immune cell deficiency of Cav‐1 on ARM function, we generated mice with specific immune cell *Cav‐1* deficiency by irradiating CD45.1 mice and reconstituting them with bone marrow cells derived from congenic WT or *Cav‐1^−/−^* mice. The efficiency of immune cell reconstitution in monocytes exceeded 70% and showed no difference between WT and *Cav‐1^−/−^* groups (Figure S12A). Similarly, obesity‐induced ARM accumulation was also diminished in *Cav‐1^−/−^* bone marrow transplant (BMT) mice (Figure S12B,C). We further established macrophage‐specific *Cav‐1* knockout mice (*MKO*) by crossing *Lyz2‐Cre* mice with *Cav‐1*‐floxed mice (*Cav‐1^f/f^*). A significant reduction of *Cav‐1* levels was observed in the ATMs isolated from *MKO* mice compared with those isolated from *Cav‐1^f/f^* mice, indicating successful deletion of *Cav‐1* (11H). Obesity‐induced ARM accumulation was significantly reduced in *MKO* mice (Figure 5I,J). Additionally, the remaining ARMs in *Cav‐1^−/−^* mice exhibited impaired adhesion capacity in comparison to WT mice (Figure 5M). Moreover, the remaining ARMs in *Cav‐1^−/−^* mice, *Cav‐1^−/−^* BMT mice and *MKO* mice accumulated less lipid compared to WT mice (Figure 5L,M; Figure S12D). In MKO mice, the remaining ARMs also exhibited reduced fatty acid uptake (Figure 5N) and lower CD36 expression (Figure 5O). Furthermore, they exhibited reduced expression of key ARM signature genes, including adhesion genes (*Cd11c*, *Cd9*, *Cdh5*) (Figure 5P) and lipid handling genes (*Fabp4*, *Pparg*, *Dgat2*, *Cavin2*, *Plin4*, *Cyp2e1*) (Figure 5Q). Taken together, these data suggest that CAV‐1 functions as a surface marker for ARMs and plays a crucial role in both the formation and maintenance of their distinctive features.

### 2.8 ARMs Are Distinct from LAMs

ARMs and LAMs shared certain phenotypic and functional features. Both subsets express high levels of CD11c and CD9, are enriched in lipid droplets, and upregulate genes involved in lipid synthesis. To determine whether ARMs and LAMs represent distinct ATM subpopulations, we reanalyzed single‐cell and single‐nucleus RNA sequencing datasets from mouse eWAT. UMAP did not cluster ARMs (*Cav‐1* ^+^) and LAMs (*Cd9* ^+^ *Trem2* ^+^), with approximately 70% of marker gene expression differs between ARMs and LAMs (Figure S13A). Flow cytometry analysis of eWAT further confirmed that the overlap between CAV‐1^+^ ARMs and CD9^+^CD63^+^ LAMs was limited to 30.4% (Figure S13B), supporting their largely nonoverlapping marker profiles.

Previous studies have shown that LAMs localize to CLSs [10]. Immunofluorescence confirmed this pattern and revealed that ARMs can also act as the dominant macrophages forming CLSs (Figure S13C). To further assess their functional distinctions, we sorted ARMs and LAMs from the eWAT of HFD‐fed (10‐week) mice and performed metabolic assays. ARMs exhibited superior adhesion (Figure S13D), fatty acid uptake (Figure S13E) and lipid storage capacity (Figure S13F) compared to LAMs, with differences in lipid uptake and processing independent of CD36 expression (Figure S13J). Within the CAV‐1^+^ macrophage population, we further subdivided cells into CD9^+^CD63^+^CAV‐1^+^ LAMs and CD9^−^CD63^−^CAV‐1^+^ ARMs. Both subsets showed similar adhesion to adipocytes (Figure S13G), whereas CD9^+^CD63^+^CAV‐1^+^ LAMs exhibited higher fatty acid uptake (Figure S13H) and lipid storage capacity (Figure S13I). Among all populations analyzed, CD9^+^CD63^+^CAV‐1^+^ LAMs displayed the highest lipid uptake and lipid‐processing capacities, indicating that while CAV‐1 defines ARMs, co‐expression of classical LAM markers further enhances lipid‐handling functions.

We next examined the ARM population in *Trem2*‐deficient mice subjected to 10 weeks of HFD. As expected, *Trem2* knockout led to a marked reduction in the proportion of LAMs among SVF macrophages (Figure S13K), consistent with previous findings [10]. However, the proportion and absolute number of ARMs remained unchanged (Figure S13L,M), indicating that ARM homeostasis is maintained through TREM2‐independent mechanisms. Collectively, these results demonstrate that ARMs and LAMs are transcriptionally, functionally, and developmentally distinct ATM populations.

### 2.9 ARMs Acquire Adipocyte RNA

**FIGURE 6:**
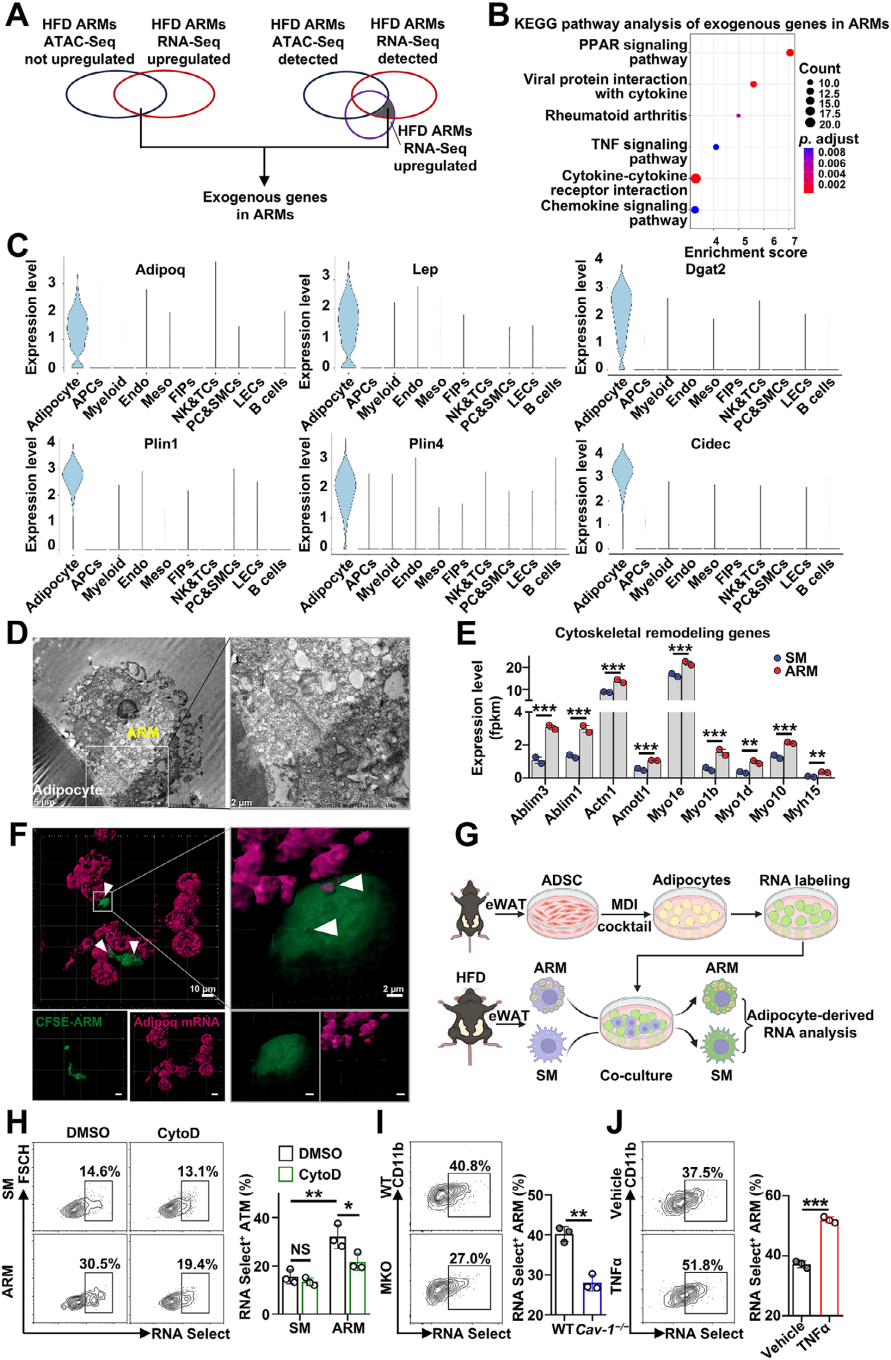
ARMs acquire adipocyte RNA. (A) Venn diagram illustrating the overlap of genes that are upregulated in RNA‐Seq but either not upregulated or not detected in ATAC‐Seq. (B) KEGG pathway analysis of exogenous genes in ARMs, depicting the six most significant pathways. (C) Violin plots of foreign ARM gene expression in different cell types from single nuclear sequencing data of eWAT. (D) Representative electron micrographs of the cell–cell contact between bead‐purified ARMs and their adhered adipocytes isolated from 8‐week HFD‐fed mice. Scale bars: 5 and 1 µm. (E) RNA‐seq gene expression levels of Cytoskeletal remodeling. (F) Representative RNAscope images showing uptake of adipocyte‐derived *Adipoq* mRNA by CFSE‐labeled ARMs after 30 min of coculture. Scale bars: 10 and 2 µm. (G) Schematic of the ex vivo assay used to quantify adipocyte‐derived RNA acquired by ARMs or SMs in (H). Mouse eWAT was digested to obtain the stromal vascular fraction (SVF), from which ADSCs were purified and differentiated into mature adipocytes. Adipocyte RNA was labeled with RNASelect and cocultured with ARMs or SMs from eWAT of HFD‐fed mice for 1 h. After trypsin digestion into a single‐cell suspension, adipocyte‐derived RNA signals in ARMs or SMs were analyzed by flow cytometry. (H) Percentage of RNASelect^+^ macrophages after coculture of RNASelect^+^ adipocytes with ARMs or SMs in the presence of the actin‐depolymerizing agent cytochalasin D (*n* = 3 per group). (I,J) Percentage of RNASelect^+^ ARMs after coculture with RNASelect^+^ adipocytes: (I) ARMs isolated from HFD *WT* and *Cav‐1^−/−^* mice; (J) ARMs after coculture with TNF‐α‐pretreated adipocytes (*n* = 3 per group). Data are expressed as means ± SEM. *p < 0.05, **p < 0.01, ***p < 0.001, NS = not significant.

CAV‐1 clearly regulates adhesion functions by anchoring various adhesion molecules and modulating their gene expression [32, 34, 35, 39, 42, 43, 44, 45, 46, 47]. While CAV‐1 participates in fatty acid uptake [38], it does not explain the alterations in lipid metabolism genes in ARM. To investigate the mechanisms by which ARMs regulate lipid metabolism genes, we integrated ATAC‐seq and RNA‐seq datasets to identify potential transcription factors. However, we struggled to identify upregulated chromatin peaks linked to RNA‐seq–upregulated lipid metabolism genes, as many lacked the corresponding peaks or showed no upregulation. This suggests these genes are likely acquired from external sources rather than being endogenous. To confirm this hypothesis, we defined genes that were upregulated in RNA‐Seq but not in ATAC‐Seq as ‘exogenous’ genes (Figure 6A). KEGG analysis revealed a focus on the PPAR signaling pathway (Figure 6B). Analysis on single‐nucleus sequencing data of eWAT revealed that many of these foreign genes are adipocyte specific genes, such as *Adipoq*, *Lep*, *Dgat2*, *Plin1*, *Plin4*, and *Cidec* (Figure 6C; Figure S14).

To investigate how ARMs acquire adipocyte RNA, we first examined whether extracellular vesicles are involved in RNA transfer between adipocytes and macrophages. Given the absence of a specific in vivo method to label adipocyte RNA, we crossed *mT/mG* mice with *Adipoq‐Cre* transgenic mice to generate adipocyte‐specific labeled mice (*mT/mG*, *Adipoq‐Cre* mice). This allowed us to investigate the impact of exosomes on the acquisition of adipocyte biomaterial by ARM or SM. Our results showed that ARMs received more tdTomato signals from adipocytes than SMs (Figure S15A). Compared to the dimethyl sulfoxide (DMSO) control, treatment with the exosome inhibitor GW4869 reduced the tdTomato signal from adipocytes in both ARMs and SMs (Figure S15C). However, the difference between the two groups was not abolished (Figure S15C,D). Importantly, the adipocyte‐derived gene differences between ARMs and SMs occur through a mechanism independent of exosomes.

Electron microscopy analysis shows the interaction between a macrophage and an adipocyte (Figure 6D), and at the ARM–adipocyte adhesion site, adipocytes extend tubular structures into ARMs (Figure S15E). The formation of tubular structures is linked to cytoskeletal remodeling [48, 49, 50]. Genes related to cytoskeletal remodeling, such as *Ablim3*, *Ablim1*, *Acrn1*, *Myo1e*, and *Myh15*, are upregulated in ARMs (Figure 6E). This suggests that ARMs may acquire material from adipocytes via cytoskeletal remodeling. RNAscope confirmed uptake of adipocyte‐derived Adipoq mRNA by ARMs at sites of cell–cell adhesion (Figure 6F). We established an ex vivo assay to measure adipocyte‐derived RNA uptake by ARMs and SMs (Figure 6G). ARMs acquired more RNA than SMs (Figure 6H). Cytochalasin D, an actin polymerization inhibitor, blocked RNA transfer to ARMs but not to SMs (Figure 6H). CAV‐1 deficiency in ARMs reduced RNA transfer, whereas TNF‐α pretreatment of adipocytes enhanced it (Figure 6I,J). These results indicate that ARMs acquire adipocyte‐derived RNA through cytoskeletal remodeling of adhesion site.

### 2.10 ARMs Are Essential for Adipose Tissue Remodeling and Glycol‐Metabolic Homeostasis

**FIGURE 7:**
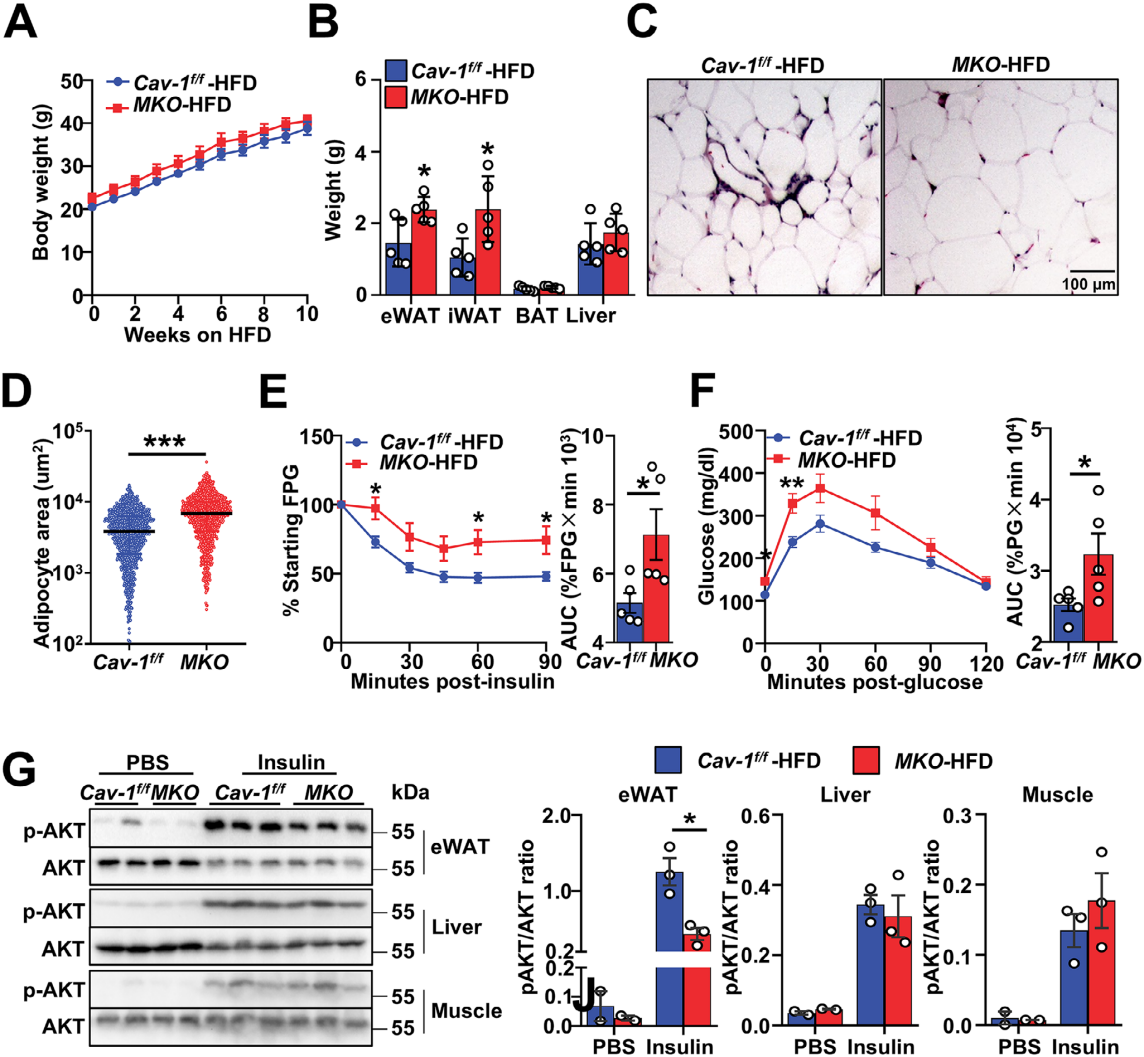
ARMs prevent adipocyte hypertrophy and glycol–metabolic disorder in vivo. (A) Body weight over time on HFD, (*n* = 5 per group). (B) Tissue weight at week 10 on HFD, (*n* = 5 per group). (C) Images of H&E stain of eWAT sections. Scale bar, 100 µm. (D) Quantification of the area of 1000 randomly selected adipocytes per genotype in H&E sections. (E) ITT in mice during week 8 of HFD feeding, (*n* = 5 per group). (F) GTT in mice during week 10 of HFD feeding, (*n* = 5 per group). (G) Immunoblots of lysates from eWAT, liver, and skeletal muscle with anti‐pAKT and anti‐AKT and quantitation of phosphorylated AKT normalized to total AKT. Data are expressed as means ± SEM. *p < 0.05, **p < 0.01, ***p < 0.001.

Finally, we sought to determine the functional importance of ARM during obesity. As a result of the critical metabolic regulatory role that CAV‐1 plays in adipocytes [51], *Cav‐1*‐deficient mice are unsuitable for investigating the in vivo functions of ARMs despite their absence of ARMs. ARM‐deficient *MKO* mice and *Cav‐1^−/−^* BMT mice were used to observe the effects. Upon HFD feeding, *MKO* mice exhibited no weight gain (Figure 7A), increased fat accumulation in eWAT (Figure 7B), substantial adipocyte hypertrophy (Figure 7C,D), worse insulin resistance and glucose intolerance (Figure 7E,F). Western blotting of eWAT, liver, and skeletal muscle revealed impaired insulin responsiveness in eWAT, as indicated by post‐insulin AKT phosphorylation (Figure 7G). These metabolic phenotypes of *Cav‐1*‐deficient chimeras were also observed under HFD conditions (Figure S16A–H) but not under ND conditions (Figure S17A–H). Together, these findings underscore the indispensable role of immune cell *Cav‐1* in maintaining adipose tissue function and metabolic homeostasis.

To further investigate the regulatory functions of ARMs from lean mice in adipose tissue homeostasis and systemic metabolism, ARMs isolated from the eWAT of lean mice were labeled with CFSE and transferred to the eWAT of obese mice. ITT, GTT, and insulin effects on target tissues were tested after ARM transplantation (Figure S18A). Transferred cells were identified through CFSE detection in ATMs. After transplantation, an increase number of CFSE^+^ ATMs was observed in the eWAT of mice that received ARM or SM injections compared to the vehicle injection group (Figure S18B), indicating successful ARM transplantation. ARM transplantation did not alter body weight, eWAT weight, and adipocyte area (Figure S18C–F); however, it significantly improved insulin sensitivity (Figure S18G), and glucose tolerance (Figure S18H). Western blotting of eWAT, liver, and skeletal muscle revealed greater insulin responsiveness in eWAT, as indicated by post‐insulin AKT phosphorylation (Figure S18I). Together, these data suggest a protective role of ARMs from lean mice in maintaining adipose tissue homeostasis and insulin sensitivity during obesity.

## 3 Discussion

In this study, we developed an approach to isolate ARMs and assess their characteristics and functions. During obesity, ARMs proliferate and increase through transfer from SM, becoming a major component of ATM. CAV‐1, a marker of ARMs, regulates their adhesion and lipid accumulation. By adhering to adipocytes, ARMs acquire adipocyte RNA, boosting their lipid uptake and storage capacity to maintain metabolic homeostasis in adipose tissue during obesity.

We optimized the method for isolating ATM. The standard protocol to isolate ATMs has been well‐established [12]. This method involves collagenase digestion followed by centrifugation to separate the upper‐layer adipocytes from the settled SVF. ATMs were then purified from SVF. In this standard protocol, collagenase degrades collagen fibers in the ECM, disrupting cell–ECM contact while preserving cell–cell adhesion [30, 31]. ARMs adhere directly to adipocytes through stable cell–cell adhesion (Figures 1B and 6D), which is resistant to collagenase digestion. In contrast, SMs interact primarily via cell–ECM adhesion, which is readily disrupted by collagenase. Therefore, Under the prevailing practice of gentle digestion to preserve adipocyte integrity [10, 13, 52], only macrophages in SVF were collected as ATMs for subsequent studies. Indeed, ARMs (CAV‐1^+^ ATMs) that are directly adhered to adipocytes have been overlooked and excluded from published single‐cell sequencing datasets of human and mouse adipose tissue derived from the SVF (Figure 5D,E). To our surprise, ARMs comprise approximately 13% of ATMs in a lean state and approximately 70% of ATMs in an obese state (Figure 1F). These results raise important considerations for ATM isolation strategies, highlighting the need to optimize protocols to ensure recovery of the full ATM compartment, including ARMs. Mechanical dissociation by vortexing enables efficient recovery of ARMs while preserving surface proteins, whereas brief trypsin digestion offers an alternative for disrupting cell–cell adhesion when surface protein preservation is less critical (Figure S1E,F) [53, 54].

Our study reveals that during HFD, adipose macrophage accumulation is mainly driven by ARMs, with SM numbers remaining stable. Three commonly used approaches have been employed to assess ATM accumulation in obesity: (1) The proportion of macrophages within the SVC; [55] (2) the total number of ATMs per tissue sample or per animal; [21, 22] (3) the number of ATMs normalized per gram of adipose tissue [21, 56, 57]. Consistent with previous studies, we observed a clear increase in SVC ATMs under HFD when measured by the first two methods (Figure S1G,H). However, using the third method, ATMs per gram of adipose tissue, yields conflicting results in the literature. Some studies report an increase in SVC ATMs with obesity [57], while others do not [56], which aligns with our findings (Figure 1E). We propose that one source of variability in SM abundance is the extent of adipocyte rupture during tissue digestion, which can release ARMs from their adipocyte‐associated position into the SVF, where they may be mistakenly counted as SMs. This effect is highly dependent on the collagenase protocol used, including mechanical force and incubation time, which vary widely across laboratories. In our study, we used a gentle digestion protocol as described in several prior publications [10, 13, 14, 56], which preserves adipocyte integrity and minimizes ARM contamination in the SVF (Figure 5B,C). Thus, rather than suggesting that previous studies “missed” ARMs entirely, our work highlights that ARMs may have been inadvertently included in SM counts in studies using harsher digestion methods. This contamination could contribute to variability and conflicting interpretations regarding ATM function in obesity.

Previous studies suggest that ATM expansion in obesity is initially driven by local proliferation, followed by increased monocyte recruitment at later stages [23, 58, 59]. To investigate the mechanisms underlying ARM accumulation, we examined the roles of proliferation, SM‐to‐ARM transition, and monocyte recruitment. During early HFD feeding, both SMs and ARMs exhibited increased proliferation, with ARMs proliferating more robustly (Figure S3A,B). This may reflect proliferation cues mediated by cell–cell adhesion [60], which depends on integrin and PI3K signaling [61, 62] —both upregulated in ARMs (Figures 2B and 3A,B). We also found that SMs can convert into ARMs in obese mice (Figure S3G), suggesting SM‐to‐ARM transition contributes to ARM accumulation. Notably, CAV‐1, the ARM marker, is upregulated in adipose tissue of obese individuals [63], potentially in response to HFD or lipopolysaccharide (LPS)‐related signaling,[64, 65] which may drive CAV‐1 expression in SMs and promote their phenotypic shift (Figure S3H,I). Collectively, increased SM‐to‐ARM conversion, rather than proliferation alone, is likely a major driver of ARM accumulation during obesity.

Our results uncover a previously unrecognized role for ARMs in maintaining adipose tissue homeostasis. ARMs are highly expressed in adhesion‐related molecules, enhancing their adhesive capacity (Figures 2 and 3). By directly adhering to adipocytes and acquiring adipocyte mRNA (Figure 6D–H), ARMs gain robust lipid uptake and storage abilities (Figure 4). This nonexosome‐dependent (Figure S15), direct RNA transfer, mediated by cytoskeletal changes at adhesion sites (Figure 6), closely resembles trogocytosis, which also involves intercellular material transfer through cell‐to‐cell adhesion and cytoskeletal remodeling [66, 67]. Our results uncover a previously unrecognized role for ARMs in maintaining adipose tissue homeostasis. ARMs are highly expressed in adhesion‐related molecules, enhancing their adhesive capacity (Figures 2 and 3). By directly adhering to adipocytes and acquiring adipocyte mRNA (Figure 6D–H), ARMs gain robust lipid uptake and storage abilities (Figure 4). This nonexosome‐dependent (Figure S15), direct RNA transfer, mediated by cytoskeletal changes at adhesion sites (Figure 6), closely resembles trogocytosis, which also involves intercellular material transfer through cell‐to‐cell adhesion and cytoskeletal remodeling [66, 67]. Consistently, the adipocyte membrane forms tubular extensions that invade adherent ARM cells, resembling classic trogocytosis (Figure 6D), and RNAscope further confirms internalization of adipocyte RNA into ARMs through these structures (Figure 6F). In CAV‐1 knockout mice, the ablation of increased lipid‐processing gene expression in the remaining ARMs, compared to WT controls (Figure 5O), suggests that CAV‐1 might regulate the formation of this structure. CAV‐1 modulates ARM adhesion to adipocytes by regulating adhesion molecule expression (Figure 5N; Figure S6E), which is also crucial for trogocytosis [67], and also influences cytoskeletal remodeling [68, 69]. Accordingly, CAV‐1–deficient ARMs exhibit impaired uptake of adipocyte RNA (Figure 6I). Although in vivo phagocytosis assays may be influenced by vessel proximity, ARMs show strong adipocyte trogocytosis but weak dextran and bacterial uptake (Figure S10), consistent with Rollins et al. [66], who reported that enhanced macrophage adhesion promotes trogocytosis while inhibiting phagocytosis. While exosome‐mediated communication between adipocytes and macrophages has been reported [70], our findings suggest, to our knowledge, the first evidence that adipocyte RNA can be transferred to macrophages via trogocytosis. TNF‐α‐stimulated adipocytes boost RNA uptake by ARMs (Figure 6J), inviting future studies into the signals driving this process and its role in metabolic disorders.

CAV‐1 is a novel surface marker of ARMs not previously reported in immune cells, as demonstrated by immunofluorescence, flow cytometry, and reanalysis of published snRNA‐Seq datasets showing its selective expression in ARMs but not SMs (Figure 5B–E); confirmed by flow cytometric analysis of adipose immune cells revealing CAV‐1 expression exclusively in ARMs (Figure S11D); and supported by its dynamic changes in CAV‐1^+^ ATMs during obesity, consistent with known ARM behavior (Figure 5F; Figure S11F,G), together establishing CAV‐1 as a specific and reliable marker of ARMs. CAV‐1 is also a population‐driven molecule of ARMs. As an essential component of lipid rafts, CAV‐1 regulates cell adhesion functions by participating in the gathering of adhesion molecules on the cell membrane [39]. It colocates with integrins, cadherins, and immunoglobulin superfamily molecules on the cell membrane [32, 42, 43, 44, 45]. Loss of Cav‐1 inhibits cell adhesion mediated by adhesion molecules [34, 35, 46, 47]. Our study demonstrates that *Cav‐1* drives the expression of cell adhesive molecules (Figure 5P). The absence of *Cav‐1* abolishes the adhesive function of ARMs (Figure 5K), leading to a reduced number of ARMs in eWAT (Figure 5G–J). In conclusion, CAV‐1 regulates ARM abundance and function and acts as a specific marker.

While ARMs share certain lipid‐accumulating features with previously described LAMs, multiple lines of evidence support that ARMs constitute a distinct subset of ATMs, rather than a variant of LAMs. Transcriptomic and flow cytometric analyses revealed that approximately 70% of marker gene expression differs between ARMs and LAMs (Figure S13A,B) [40], indicating substantial heterogeneity. ARMs and LAMs also display distinct distributions within adipose tissue: CLSs dominated by ARMs were identified (Figure S13C, lower panel). Functional assays further confirmed that ARMs exhibit superior fatty acid uptake and neutral lipid accumulation compared to LAMs (Figure S13E,F), and ATM coexpression of classical LAM markers (CD9, CD63) with the ARM marker CAV‐1 further enhances lipid‐handling capacity (Figure S13H,I). Importantly, LAM accumulation is dependent on TREM2 signaling, as previously reported by Jaitin et al. and confirmed in our study (Figure S13K) [10]. In contrast, ARM abundance was unaffected in *Trem2* knockout mice (Figure S13L,M), suggesting that ARMs are maintained independently of the TREM2 pathway. Collectively, these findings demonstrate that ARMs represent a functionally, spatially, and developmentally distinct ATM population, separate from LAMs.

Our study identifies ARMs as an important subset in maintaining metabolic homeostasis. It is likely that the surge in ARM reflects an adaptive response to high‐fat diet stimuli. Specifically, during the early stages of HFD‐induced metabolic stress—when insulin resistance is driven by lipid overload [22, 71] —ARMs expand and enhance their lipid uptake and storage capacity through direct adhesion to adipocytes and cytoskeleton remodeling–mediated acquisition of adipocyte‐derived mRNA (Figures 4 and 6). This allows ARMs to buffer excess free fatty acids by promoting triglyceride synthesis and lipid storage (Figure 4D–J), which helps limit adipocyte hypertrophy and preserve insulin sensitivity (Figure 7; Figures S16 and S18). However, this protective effect appears to be time‐limited. With prolonged HFD feeding, lipid burden may exceed the storage capacity of ARMs, leading to functional exhaustion and metabolic decompensation (Figure S7A). At this stage, both ARMs and SMs begin to exhibit low‐grade pro‐inflammatory profiles (Figure S7B,C), contributing to the chronic inflammatory environment characteristic of advanced obesity. Therefore, ARMs initially act to maintain metabolic homeostasis by mitigating lipid‐induced stress in adipose tissue. Their depletion—such as in CAV1‐deficient reconstitution experiments—disrupts this buffering mechanism, leading to exaggerated adipocyte expansion and impaired insulin sensitivity. This highlights the temporally dynamic and context‐dependent roles of distinct macrophage subsets in obesity. Understanding the long‐term metabolic roles of ARMs and how their lipid‐handling capacity is maintained during chronic obesity may provide critical insights into the transition from metabolically healthy to pathological obesity, as observed clinically.

Our study fills a critical gap in the current understanding of ATMs by identifying and characterizing ARMs as a distinct subset. By adhering to adipocytes, ARMs acquire adipocyte‐derived RNA, which enhances their lipid uptake and storage capacity, thereby supporting metabolic homeostasis during obesity. Both the development and function of ARMs are dependent on their surface marker, CAV‐1. Notably, ARMs are not confined to adipose tissue; similar adhesive interactions with hepatocytes and tumor cells were observed in the liver and tumors, respectively (Figure S2). ARMs may influence tissue homeostasis and disease progression in a manner analogous to their role in adipose tissue. The study presents novel insights into the domain of tissue macrophages and emphasizes the importance of conducting a thorough investigation into the functions of ARMs beyond adipose tissues.

### 3.1 Limitations of The Study

Owing to the absence of adipocyte‐specific RNA tracing tools in vivo, direct evidence of RNA transfer at the ARM–adipocyte interface in vivo could not be captured. Nonetheless, this process was recapitulated in vitro by labeling adipocyte RNA and co‐culturing with ARMs, with RNAScope confirming RNA transfer. Further clinical studies should investigate the correlation of ARMs with weight control and metabolic parameters in obese or diabetic patients.

## 4 Experimental Section

### 4.1 Mice

WT mice (C57BL/6J) were purchased from Slac Laboratory Animal Inc (Shanghai, China, >5 generations), and *Cav‐1* KO mice were purchased from Jackson Laboratory (Stock NO. 007083, Bar Harbor, ME, USA, >7 generations). *Trem2* knockout (*Trem2^−/−^*) mice were constructed from Cyagen Biosciences, Inc. (Suzhou, China, >7 generations). *mT/mG* mice were purchased from Gempharmatech (Stock NO. T006163, Suzhou, China, maintained >4 years), and *Adipoq‐Cre* mice were purchased from Jackson Laboratory (Stock NO. 028020, Bar Harbor, ME, USA, maintained >4 years). *Cav‐1‐floxed* mice were purchased from SHANGHAI MODEL ORGANISM (Stock NO. NM‐CKO‐2116185, Shanghai, China, >5 generations), and *Lyz2‐Cre* mice were purchased from Jackson Laboratory (Stock NO. 004781, Bar Harbor, ME, USA, maintained >7 years). All mice were on a C57BL/6 background and kept in the specific pathogen free animal room, maintaining a constant temperature and 12 h light‐dark cycle. The WT and *Cav‐1* KO mice were crossed, and the F1 heterozygous mice were used to generate WT and *Cav‐1* KO littermates for experimental research. The *mT/mG* and *Adipoq‐Cre* mice were crossed, and the F1 heterozygous mice were used to generate *mT/mG*, *Adipoq‐Cre* mice for experimental research. At 6 weeks of age, male mice were fed a ND1(0% fat, MD17121) (Medicience Ltd, Jiangsu, China) or a HFD (60% fat, D12492) (Research Diets, New Brunswick, NJ). For bone marrow chimera experiments, mice were given a sublethal dose of total body irradiation (9.5 Gy). 6 h later, mice were transplanted with 1 × 10^7^ bone marrow cells. Mice were allowed 6 weeks for reconstitution before HFD treatment. Reconstitution efficiency was tracked using blood monocyte with the congenic marker CD45.1. All animal studies were performed in accordance with procedures approved by the Central South University Animal Care and Use Committee.

### 4.2 Human Subjects

Human VAT samples were procured from two distinct cohorts: morbidly obese individuals who met the specific recruitment criteria for bariatric surgery, and individuals undergoing nonacute surgical procedures, such as cholecystectomy, as part of their regular scheduled surgeries. This investigation was conducted in strict accordance with the principles of the Declaration of Helsinki and was ethically approved by the Ethics Committee of the Second Xiangya Hospital of Central South University (Approval No. LYF2022207). Written informed consent was obtained from all participants, and they fell within the age range of 18 to 56 years. The tissue samples, each weighing 0.2 g, were washed and fixed upon removal from the patients.

### 4.3 Adipose Tissue Fractionation

Adipose tissues were rinsed in Dulbecco's modified MEM (DMEM), minced to about 8 mm^3^ fragments, and subjected to thorough washing with PBS, followed by centrifugation. Subsequently, the adipose tissue was subjected to collagenase digestion, and digested with 1 mg/mL type II collagenase and 1% bovine serum albumin in DMEM for 30 min at 37°C with gentle agitation. After complete digestion of the tissue fragments into a cell suspension, the sample was centrifuged at 500 g for 5 min at room temperature, separating the adipocyte‐rich upper layer from the SVF‐containing precipitate. The adipocyte layer was resuspended with PBS containing 2 mm ethylenediaminetetraacetic Acid (EDTA) in a new 15 mL tube, and filtered through a 100 µm nylon mesh. After centrifugation at 500 g for 5 min, adipocytes were collected to 1.5 mL EP tube and suffered to a vortex oscillation with a 1 mm beads until white adipocyte lysis to lipid. The mixture was then added to the liquid collected after filtering the adipocyte suspension, followed by centrifugation at 500 g for 5 min to collect the bottom precipitate, representing the adipocyte layer cells. The precipitate containing SVF were resuspended in PBS containing 2 mm EDTA and then filtered through a 100 µm nylon mesh. After centrifugation at 500 g for 5 min, bottom precipitate was collected, which represents the stromal vascular cells. Subsequently, two group of cells were incubated in red blood cell lysis buffer for 5 min on ice, then washed with PBS containing 2 mm EDTA and collected by centrifugation at 500 g for 5 min. The resultant adipocyte layer cells and SVF were subjected to downstream analysis.

### 4.4 Liver Fractionation

The liver tissue underwent in situ digestion using previously established methods [72]. In brief, after euthanizing the mice, we opened the abdominal cavity and flushed the liver with HBSS solution injected through the inferior vena cava until the blood flow was clear. Following this, a digestion solution containing 100 U/mL type IV collagenase and 10 µg/mL DNase‐1 was perfused. After the liver scaffold collapsed, the liver was extracted, the liver capsule torn open, and the liver gently squeezed to release the liver cells. After filtration through a 100 µm mesh, cells were centrifuged at 50 g for 3 min at 4°C, washed 3 times with DMEM medium, and subjected to percoll separation to collect the supernatant for obtaining single nuclear cells. The precipitated liver cells were subjected to trypsin digestion for 30 s, followed by another round of percoll separation to obtain single nuclear cells. These two cell fractions were collected and prepared for further analysis.

### 4.5 Tumor Fractionation

The MC38 cells were harvested, resuspended at a concentration of 1 × 10^5^ cells/50 µL in PBS, and then injected subcutaneously into 6‐week‐old female C57BL/6 mice. After 3 weeks, tumor tissues were dissociated following established protocols [73]. In brief, tissues from euthanized tumor‐bearing mice were minced and digested at 37°C in DMEM with 1 mg/mL type IV collagenase, 10 µg/mL DNaseI, and 2% fetal bovine serum (FBS) for 30 min. After filtration and centrifugation at 500 g for 5 min at 4°C, the bottom pellet underwent Percoll separation to obtain single nuclear cells and the tissue cell layer. The collected tissue cell layer was subjected to a 50 s trypsin digestion along with the single nuclear cell layer for further analysis.

### 4.6 Adipose Tissue Dissociation for Marker Detection

To accurately determine the distribution of CAV‐1^+^ macrophages in both the SVF and the adipocyte layer, we employed a more stringent tissue dissociation method. We first minced the adipose tissue, then washed it with PBS and centrifuged to collect the adipose tissue for digestion. 1 gram of adipose tissue fragments was added to a 15 mL centrifuge tube along with 7 mL of 1 mg/mL type II collagenase and 1% bovine serum albumin in DMEM for 30 min at 37°C with 40 rpm agitation. After most of the tissue fragments were digested into a cell suspension, the centrifuge tube was removed and allowed to stand at room temperature for 5 min to let the adipocytes float to the top layer. The upper layer containing the crude adipocytes was aspirated, filtered through a 100 µm nylon mesh, and centrifuged. Adipocytes were collected to 1.5 mL EP tube and suffered to a vortex oscillation with a 1 mm beads until white adipocyte lysis to lipid, followed by centrifugation at 500 g for 5 min to collect the bottom precipitate, representing the adipocyte layer cells. The remaining adipocytes‐free liquid postdigestion was filtered and centrifuged to obtain the SVF. These two cell fractions were collected and prepared for FCM analysis.

### 4.7 Flow Cytometry and Cell Sorting

Flow Cytometry analysis was performed using murine single‐cell suspensions. Cells were Fc receptor–blocked with anti‐CD16/32 (1:100, 101302, Biolegend) prior to staining with antibodies against CD45 (1:200, 103108, Biolegend), CD11b (1:200, 101228, Biolegend), F4/80 (1:200, 123114, Biolegend), CD9 (1:200, 124806, Biolegend), CD63 (1:200, 143905, Biolegend), CD45.1 (1:100, 110728, Biolegend), CD45.2 (1:100, 109824, Biolegend), Ki67 (1:200, 652410, Biolegend), and BODIPY (0.2 µg/mL, D3922, Thermo Fisher Scientific). Cells were also stained with viability dyes including propidium iodide (1:200, 421301, Biolegend), Zombie NIR (1:100, 423106, Biolegend), or DAPI (0.2 µg/mL, 4083, Cell Signaling Technology). For CAV‐1 flow cytometry analysis, live cells were first blocked with CD16/32 and FBS, stained for surface markers and viability dye, then fixed and permeabilized according to the BD Fixation/Permeabilization Kit protocol, followed by staining with anti–CAV‐1 (1:200, D46G3, Cell Signaling Technology) and a fluorescence‐conjugated secondary antibody (1:200, Invitrogen, Cat# A‐11034). Cells were analyzed using a BD FACSAria III, and data were processed with FlowJo software (version 10.4; Tree Star). Cell sorting based on surface marker staining was performed using either a S3e cell sorter (Bio‐Rad) or BD FACSAria III.

### 4.8 Cell Culture

ARMs and SMs were beads purified (Anti‐F4/80 MicroBeads UltraPure, Miltenyibiotec, 130‐110‐443) from adipocyte layer cells and SVF respectively. Cells were resuspended in RPMI 1640 plus 10% FBS, 100 IU/mL of penicillin, 100 mg/mL of streptomycin solution), at 5% CO2 and 37°C in a humidified cell incubator. After seeding, the medium was changed to remove nonadherent cells after 24 h. For in vitro adipogenesis, Adipose‐derived stem cells (ASCs) were purified using a serial passaging method as previously described [74]. Briefly, adipose tissue was digested with collagenase, and the SVF was collected by filtration and centrifugation. SVF cells were cultured in F12/DMEM medium supplemented with 10% FBS and 1% penicillin/streptomycin. Medium was changed every 2–3 days, and cells were passaged at approximately 80% confluence using trypsin at a 1:2 split ratio. Cells were expanded to passage 2–3 to obtain a purified ASC population. Purified ASC were used for adipogenesis. ASCs were incubated in a differentiation induction cocktail for 72 h: 0.5 mm 3‐isobutyl‐1‐methylxanthine (IBMX, 15879‐1G, Sigma), 1 µm dexamethasone (D4902, Sigma), 170 nm insulin (12585014, Gibco), followed by maintenance treatment (10 µg/mL insulin) every 48 h. After 7 days maintenance, cells were subjected to downstream assays. The MC38 colon adenocarcinoma cells were cultured at 37°C with 5% CO2 in DMEM supplemented with 10% FBS, 2 mm glutamine, 0.1 mm nonessential amino acids, 1 mm sodium pyruvate, 10 mm HEPES, and 1 × penicillin/streptomycin.

### 4.9 H&E and Immunofluorescence Staining

Paraffin‐embedded tissue blocks from mice were sectioned at 7 µm thickness, deparaffinized, rehydrated, and stained with hematoxylin and eosin. Adipose tissue sections were heated at 60°C for 45 min, deparaffinized using xylene, and subsequently hydrated with 100%, 95%, and 70% ethanol for 5 min at each step. For antigen retrieval, slides were placed in a container filled with boiled AR6 buffer, then placed in the microwave for another 15 min at 20% power. After retrieval, slides were placed in the AR6 buffer to cool down to room temperature. To minimize nonspecific staining, sections were blocked with 5% bovine serum albumin (BSA) in PBST for 1 h at room temperature. For mouse tissue sections, incubation with the primary antibody (1:200 CAV‐1, Cell Signaling Technology, Cat#D46G3) was performed overnight at 4°C, followed by incubation with a fluorescence‐conjugated secondary antibody (1:200, Invitrogen, Cat# A‐11012) for 60 min at room temperature. Then, the sections were incubated with another primary antibody (1:200 F4/80, Abcam, Cat# AB6640) at 4°C overnight, followed by incubation with a fluorescence‐conjugated secondary antibody (1:200, Abcam, Cat# ab150157) for 60 min at room temperature. For human tissue sections, incubation with the primary antibody (1:200 CAV‐1, Cell Signaling Technology, Cat#D46G3) was performed overnight at 4°C, followed by incubation with an HRP‐secondary antibody, and then signal amplification with a fluorescence‐conjugated antibody (1:200, Perkinelmer, 2494755). After incubation, the antibodies were stripped by microwave treatment in AR6 buffer. Then, followed by another primary antibody incubation (1:200 CD68, Cell Signaling Technology, Cat# D489C) at 4°C overnight. Repeat the incubation with HRP and another fluorescence‐conjugated antibody (1:200, Perkinelmer, 2494755). After stripping the antibodies by microwave, slides were stained with DAPI and examined under a fluorescence microscope.

For immunofluorescence of CAV‐1 in ARMs and SMs, ARMs and SMs from HFD‐fed mice were isolated by magnetic bead sorting and cultured overnight in confocal dishes. Cells were fixed with paraformaldehyde, permeabilized with Triton X‐100, blocked with 2% FBS, and incubated with anti–CAV‐1 (1:200, Cell Signaling Technology, Cat# D46G3) overnight at 4 °C. After three PBS washes, cells were incubated with a fluorescence‐conjugated secondary antibody (1:200, Invitrogen, Cat# A‐21070) for 1 h at room temperature, followed by three PBS washes. Nuclei were stained with 0.1% DAPI for 10 min, washed 3 times with PBS, and imaged on a confocal microscope (Zeiss LSM900).

For immunofluorescence of suspended adipocytes, 50 µL of adipocytes obtained after digestion of adipose tissue were diluted in 50 µL of FACS buffer to prepare a cell suspension. To this cell suspension, 1 µL of anti‐mouse CD16/32 antibody was added and incubated at room temperature for 7 min. Subsequently, anti‐mouse PE/Dazzle 594‐F4/80 antibody was added for staining, and the mixture was incubated in the dark at room temperature for 15 min. After washing, cells were incubated in 1 mL of FACS buffer containing 0.1 mg/mL of BODIPY and 0.1 mg/mL of HOECHST, followed by another incubation in the dark at room temperature for 15 min. After washing, the cells were spread onto a 12‐well plate for observation and photography under a fluorescence microscope.

For immunofluorescence analysis of suspended hepatocytes, 20 µL of hepatocytes obtained after digestion were diluted in 80 µL of FACS buffer to create a cell suspension. To this suspension, 1 µL of anti‐mouse CD16/32 antibody was added and incubated at room temperature for 7 min. Subsequently, anti‐mouse PE/Dazzle 594‐F4/80 antibody was introduced for staining, and the mixture was incubated in the dark at room temperature for 15 min. After washing, cells were fixed, stained with DAPI, and resuspended in 1 mL of FACS buffer before being plated onto a 12‐well plate for observation and imaging under a fluorescence microscope.

For immunofluorescence of suspended tumor cells, the cell staining protocol follows the scheme published in the previous article. 20 µL of tumor cells obtained after digestion were diluted in 80 µL of FACS buffer to create a cell suspension. To this suspension, 1 µL of anti‐mouse CD16/32 antibody was added and incubated at room temperature for 7 min. Subsequently, anti‐mouse PE/Dazzle 594‐F4/80 antibody and anti‐mouse FITC CD45 antibody were introduced for staining, and the mixture was incubated in the dark at room temperature for 15 min. After washing, cells were fixed, stained with DAPI, and resuspended in 1 mL of FACS buffer before being plated onto a 12‐well plate for observation and imaging under a fluorescence microscope.

For immunofluorescence staining of TREM2 and CAV‐1 in CLS, obese mice were euthanized and transcardially perfused with heparinized saline (1 × 10^4^ U/L), followed by 1% paraformaldehyde. eWAT was harvested, postfixed in 1% paraformaldehyde for no longer than 15 min, cut into ∼2 mm^3^ pieces, washed with PBS, and blocked with 2% FBS and anti‐CD16/32 antibody for 1 h at room temperature. Tissues were incubated overnight at 4°C with anti‐TREM2 antibody diluted in PBS containing 2% BSA, washed 3 times with PBS, and incubated with a fluorescence‐conjugated secondary antibody (1:200, Invitrogen, Cat# A‐21070) for 1 h at room temperature, followed by three additional PBS washes. Samples were then incubated overnight at 4°C with anti–CAV‐1 and anti–F4/80 PE‐Dazzle594 antibodies, washed 3 times with PBS, and incubated with a fluorescence‐conjugated secondary antibody (1:200, Invitrogen, Cat# A‐11034) for 1 h at room temperature, followed by three PBS washes. Nuclei were counterstained with 0.1% DAPI for 10 min, washed 3 times with PBS, and tissues were placed in confocal dishes and imaged using a confocal microscope (Zeiss LSM900).

### 4.10 Western Blot

Tissues were lysed in RIPA buffer (Beijing Dingguo Changsheng Biotech, Cat#WB‐0072) with protease inhibitor phenylmethylsulfonyl fluoride (PMSF) (Beyotime, Cat#ST505) and cOmplete EDTA‐free (Sigma‐Aldrich, Cat#4693132001). The protein concentration was determined with Pierce Microplate BCA Protein Assay kit (Thermo scientific, Cat#23252). 30 µg of total protein were separated in 10% sodium dodecyl sulfate (SDS)–polyacrylamide gel electrophoresis and transferred to PVDF membrane (Merck Millipore, Burlington, MA). The membranes were blocked in 5% BSA in PBS for 1 h at room temperature and then incubated overnight at 4°C with the following primary antibodies. The membrane was washed with TBST 3 times for 15 min each. Incubate with antiHRP secondary antibody (Boster, Cat#BA1054) diluted 1:5000 in 2% BSA for 1 h at room temperature on a shaker. The membrane was washed as previously. Clarity Western ECL Substrate (Bio‐rad, Cat#170‐ 5061) was applied and exposed to film. Bio‐Rad Image Lab and Image‐Pro Plus software were used for analysis.

### 4.11 Edu Incorporation Assay

The EdU incorporation assay was conducted following the manufacturer's instructions using the Click‐iT EdU Assay Kits (Invitrogen, C10418). Mice received an intraperitoneal injection of 20 µg of EdU per gram of body weight 16 h prior to euthanasia. The Click‐iT reaction was performed on adipose tissue samples and subsequently analyzed using FCM.

### 4.12 Bone Marrow Transplantation

9‐week‐old C57BL/6J mice were administered water acidified with hydrochloric acid (pH 2.8–3.0) containing neomycin (1 g/L) for 7 days before irradiation. These mice were then exposed to a lethal dose of 9.5 Gy of irradiation. Bone marrow cells were obtained from male *Cav‐1^−/−^* mice or age‐ and sex‐matched C57BL/6J mice by flushing the femurs, tibias, and humerus with 1640 medium. 6 h after irradiation, the mice received an intravenous injection of 5 × 10^6^ bone marrow cells. Subsequently, the mice were housed in an environment with water acidified with hydrochloric acid (pH 2.8–3.0) containing neomycin (1 g/L) and were provided sterilized food for 4 weeks. After 6 weeks posttransplantation, the mice were either fed a HFD or continued on a ND for an additional 10 weeks.

### 4.13 ATMs Transplantation

C57BL/6J male mice were anesthetized with isoflurane, and surgeries were performed using sterile techniques. A total of 1.5 × 10^5^ lean ARMs or SMs (donor cells) were resuspended in 100 µL DMEM mixed with Matrigel (356237, Corning) at a 7:1 ratio on ice. These ATMs or vehicle were then injected into the bilateral visceral fat pads (50 µL per pad). Macrophages or vehicle transplantation into adipose tissue was performed using multiple injection sites per depot, following a previously reported protocol [75]. Briefly, anesthetized mice underwent a midline abdominal incision, and one side of the eWAT was gently exteriorized. Cells suspended in Matrigel/1640 medium were injected at three sites along the longitudinal axis of each fat pad, avoiding blood vessels. Cells were delivered slowly while withdrawing the needle to minimize leakage. The contralateral fat pad was injected in the same manner. The tissue was then returned to the abdominal cavity, the incision closed, and saline was administered subcutaneously for hydration. Two days after surgery, ITT was performed. Five days after surgery, GTT was performed. Eight days after surgery, after fasting for 6 h, mice were intraperitoneally injected with insulin (4 U per kg body weight) or PBS. eWAT, liver, and skeletal muscle were harvested 15 min after injection and collected for Western blot.

### 4.14 RNA Isolation, qRT‐PCR and Bulk RNA‐Seq Library Preparation

Total RNA of ARM/SMs that were purified via FACS sorting was extracted by Trizol (Life Technologies, Cat#15596026) according to the manufacturer's protocol. The RNA was reverse transcribed using a Revert Aid First Strand cDNA Synthesis Kit (Thermo scientific, Cat#K1622). RT‐qPCRs were run using SYBR Green master mix (YESEN) on Applied Biosystems ViiA 7 Real‐Time PCR System. Normalized mRNA expression for mouse samples was calculated using *bactin* as the reference gene. Relative mRNA expression was calculated using the ΔΔCt method. All primers were designed by tsingke Biotech, and the sequence of primers were listed in Table S1.

Total RNAs of ARMs and SMs were isolated and followed by RNA‐seq analysis in the company (The Beijing Genomics Institute, Shenzhen, China) using the Illumina high‐throughput sequencing platforms. The differential expression of genes was selected by Log2FC > 0.6 or Log2FC < −0.6 and *p* < 0.05.

### 4.15 ATAC‐Seq Library Preparation

ATAC‐seq was conducted following a protocol adapted from Buenrostro et al. [76] Briefly, ARM/SMs that were purified via FACS sorting were first washed with PBS and then lysed using a lysis buffer composed of 1 m Tris·Cl (pH 7.4), 5 m NaCl, 1 m MgCl2, and 10% Nonidet P‐40 in nuclease‐free H_2_O, with gentle agitation. Following this, the lysed samples were treated with Tn5 transposase. DNA was purified (Qiagen Min Elute Reaction Cleanup Kit) and amplified with 1X NEB Next High‐Fidelity PCR Master Mix (New England Biolabs, MA) using barcoded primers. DNA library was purified with Min Elute PCR Purification Kit (Qiagen). Libraries were sequenced using the Illumina Novaseq 6000 platform. Reference cistromes from BMDM were obtained from cistrome.org for the validation of the motif search results (H3K27ac, GSM1964730; H3K4me1, GSM1022290; AP‐1, GSM2974799; Atf3, GSM2663858).

### 4.16 Proteomic Library Construction

ARMs and SMs were isolated by FACS sorting, washed, snap‐frozen, and sent to QLBio for protein extraction and library construction. Proteins were extracted in 8 m urea with protease inhibitors, quantified, reduced with dithiothreitol, digested overnight with trypsin, and desalted. Peptides were fractionated by high‐pH reversed‐phase high‐performance liquid chromatography into six fractions, dried, and reconstituted in 0.1% formic acid with indexed retention time standard peptides. A proteomic library was generated using data‐independent acquisition mode.

### 4.17 Secondary Analysis of scRNA‐seq and snRNA‐seq Datasets

To assess CAV‐1 expression in monocytes and ATMs from mouse eWAT, single‐cell data and metadata from the GSE128518 dataset were downloaded from the GEO database, and the processed single‐nucleus RNA‐seq RDS file was obtained from https://github.com/JesperGrud/snRNAseq_eWAT for downstream analysis. We extracted matrix data corresponding to annotated monocyte/macrophage subpopulations, reconstructed Seurat objects, and performed standard dimensionality reduction and clustering. CAV1 expression was visualized on UMAP plots of both the merged dataset and separately analyzed scRNA‐seq and snRNA‐seq data. Cells with CAV1 expression values >0 were defined as CAV1‐positive, and those with values = 0 as CAV1‐negative. Proportions were calculated and presented as stacked bar plots.

To assess CAV1 expression in monocytes and ATMs from human WAT, The single‐cell and single‐nucleus RNA sequencing RDS files of WAT from the study *“A single‐cell atlas of human and mouse white adipose tissue”* (Nature) were downloaded from https://gitlab.com/rosen‐lab/white‐adipose‐atlas. Data analysis was performed following the same procedure as previously described.

### 4.18 Adhesion Assay

For attachment assay in culture‐dish, magnetically purified ARMs/SMs, at a density of 5 × 10^4^ cells per well, were added to a matrigel‐coated 96‐well plate. Two wells in each group were designated as controls for total cell counts without washing. After seeding cells into three additional wells for 30 min, the adhesion group was washed twice with PBS, and any residual liquid was removed by aspiration. Subsequently, the cells were fixed with 4% paraformaldehyde. DAPI staining was performed, and cells were counted using fluorescence microscopy. For attachment assay on adipocytes, 1 × 10^5^ CFSE‐labeled ARMs/SMs were added to mature adipocytes induced in a 24‐well plate. A control group was established without washing. After seeding cells into three wells for 30 min, the adhesion group was washed twice with PBS, and any residual liquid was removed by aspiration. Then, cells were fixed with 4% paraformaldehyde. DAPI staining was performed, and cells were counted using fluorescence microscopy.

### 4.19 Measurement of Fatty Acid Uptake, Lipid Synthesis and Fatty Acid Beta‐Oxidation

Magnetically purified ARMs/SMs, at a density of 5 × 10^4^ cells per well, were added to a 96‐well plate, followed by overnight cell culture. For the lipid uptake assay, cells were washed twice with PBS after a 30 min incubation in FBS‐free culture medium. Then, 10 µm BODIPY FL. C12 in FBS‐free medium was added, and the cells were incubated for 25 min. Following incubation, the cells were washed again with PBS, fixed, and stained with DAPI. Lipid uptake was assessed using fluorescence microscopy. For lipid synthesis assay, oleic acid was solubilized using KOH and incubated at 37°C in sterile water with BSA (devoid of free fatty acids) for 30 min. The cell culture medium was supplemented with oleic acid to a final concentration of 400 µm, while the control group received an equivalent amount of BSA devoid of free fatty acids. The cells were incubated for 4 h. After the cell treatment, macrophages were detached from the culture plate by digesting with PBS containing 5 mm EDTA at 4°C for 40 min, with intermittent shaking every 5 min until the cells detached from the culture plate. The neutral lipid content within the cells was assessed using BODIPY fluorescence via FCM. FAO of ARMs and SMs was determined using a FAO colorimetric assay kit according to the manufacturer's instructions (Elabscience).

### 4.20 Macrophage Phagocytosis Assays

For macrophage dextran phagocytosis assay: 100 µg/mL FITC‐labeled 70 kDa dextran was added into macrophage complete growth medium. The mixture was then incubated for 30 min. After removing the medium, cells were washed twice with PBS. Subsequently, the cells were fixed and stained with DAPI. Phagocytosis was observed and captured under a fluorescence microscope.

For macrophage *E. coli* phagocytosis assay: *E. coli* bacteria were cultured in sterile LB medium at 37°C and 220 rpm overnight. Afterward, 1 mL of growing *E. coli* culture was collected and centrifuged at 4000 g for 5 min at room temperature to remove the supernatant. The bacterial pellet was resuspended in 1 mL of PBS and washing for 3 times. Subsequently, the bacteria were resuspended in 1 mL of PBS containing 10 µm CFSE and incubated in the dark at 37°C and 220 rpm for 1 h in an orbital shaker. After incubation, the bacteria were washed again 3 times as previously described. The bacteria were heat‐inactivated at 65°C for 15 min and stored at 4°C in the dark. 1 × 10^6^ CFSE‐labeled *E. coli* bacteria were added and gently mixed with the cells, then incubated for 1 h. After removing the medium, the cells were washed twice with PBS. Subsequently, the cells were fixed and stained with DAPI. Phagocytosis was observed and captured under a fluorescence microscope. For in vivo dextran phagocytosis assay: mice were intravenously injected with FITC‐labeled 70 kDa dextran at a dosage of 18 mg/kg, dissolved in PBS with a volume of 300 µL. After 30 min, mice were euthanized, and their eWAT was collected. The collected tissue was subjected to collagenase digestion and subsequently macrophage phagocytosis analyzed using FCM.

### 4.21 Lipid Profiling

Following a 14 h fasting period, blood samples were collected from the ocular region of mice. The concentrations of triglycerides, cholesterol, high density lipoprotein (HDL), low denisty lipoprotein (LDL), and free fatty acids were determined using the Kenshin‐2 kit (Medtechnica) on the SPOTCHEM EZ sp‐4430 analyzer (Arkray).

### 4.22 Electron Microscope Imaging

eWAT from HFD mice was digested with collagenase and filtered through a 100 µm mesh into a 50 mL tube. After standing for 3 min at room temperature to allow adipocytes to float, the lower liquid was removed. The adipocytes were washed with MACS buffer (PBS with 2 mm EDTA and 0.1% BSA) 3 times. Then, 1 mL of adipocytes was incubated with 2 mL of F4/80 antibody (Biotin anti‐mouse F4/80, Biolegend, 123106) (1:200 in MACS buffer) for 15 min at room temperature. After washing twice with MACS buffer, cells were incubated with Biotin magnetic beads (CELLection Biotin Binder Kit, Invitrogen, 11533D) for 15 min, and macrophages with bound adipocytes were captured using a magnet. Finally, cells were fixed with 2.5% glutaraldehyde for 15 min and embedded in 4% agarose. The specimens were sliced into 1 × 1 × 3 mm^3^ in size and double‐fixed in 2.5% glutaraldehyde solution with Millonig's phosphate buffer (pH = 7.3) and shipped overnight at ambient temperature. In sample preparations, the samples were washed 3 times at 10 min intervals with Millonig's phosphate buffer; incubated for1 h in 1% osmium tetroxide and washed 3 times at 10 min intervals with Millonig's phosphate buffer. Dehydration of the samples were carried out at room temperature in a graded series of 50%, 70%, and 90% acetone at10 min intervals for each step followed by 100% acetone twice at 15 min intervals. Sample resin soaking and embedding process was the specimens in 1:1 mix of acetone: resin for 12 h and 100% resin to polymerize overnight at 37°C. Sample resin solidifying process was the specimens 100% resin to polymerize overnight at 37°C and then12 h at 60°C. 50–100 nm ultrathin sections of specimens were made with an ultramicrotome and a diamond knife. After 3% uranyl acetate and lead nitrate double staining, the specimens were examined and photographed on a Hitachi HT‐7700 electron microscope.

### 4.23 Coculture Experiment

eWAT from ND mice was digested with collagenase to obtain the SVF, which was then purified to yield ADSCs. ADSCs were induced to differentiate into adipocytes. On day 7 of differentiation, the adipocytes were washed with PBS and digested with 0.5 mg/mL collagenase at 37°C for 10 min, followed by a 1 min digestion with 0.25% trypsin. Detached cells were centrifuged, washed, resuspended in differentiation medium, and seeded into 24‐well plates. After 24 h, the medium was replaced with growth medium. For HFD 6 W mice, eWAT was digested with collagenase, and magnetic beads were used to isolate the ARM and SM populations. Macrophages and adipocytes were pretreated with DMSO or 50 µm Cytochalasin D (Topscience, T3229) for 1 h. For TNF‐α stimulation, adipocytes were treated with TNF‐α (20 ng/mL) for 16 h prior to coculture. Adipocytes were then incubated with 1 µm SYTO RNASelect (Invitrogen, S32703) for 20 min, followed by 3 times PBS washing. Macrophages were treated with PBS containing 5 mm EDTA at 4°C for 10 min, then collected by centrifugation. Macrophages and adipocytes were then cocultured in the presence of 50 µm Cytochalasin D for 1 h. The cells were washed, digested with 0.25% trypsin, and collected for flow cytometry analysis.

### 4.24 RNA In Situ Hybridization

ADSCs were differentiated into adipocytes in confocal dishes as described above. On day 7 of differentiation, adipocytes were washed with PBS and briefly digested with collagenase (0.5 mg/mL, 37°C, 5 min) to remove deposited extracellular collagen while avoiding cell detachment. After two PBS washes, adipocytes were cocultured with CFSE‐labeled ARMs isolated from HFD‐fed mice for 30 min. Cells were then washed and fixed with 4% paraformaldehyde for 30 min, followed by in situ hybridization for *Adipoq* mRNA using the PinpoRNA RNA in situ hybridization kit according to the manufacturer's instructions. Fluorescent signals were labeled using tyramide signal amplification substrates, and images were acquired using a confocal microscope (Zeiss LSM900). 3D reconstruction was performed using IMARIS 9.0.1.

### 4.25 Exosome Inhibition

To inhibit sEV (including exosome) biogenesis, neutral sphingomyelinase‐2 inhibitor GW4869 (2.5 mg/kg/2d) was administered via intraperitoneal injection. One week after GW4869 administration, plasma and eWAT were collected. The same volume of dimethyl sulfoxide‐treated mice was used as control.

### 4.26 GTT and ITT

For glucose tolerance tests, mice were subjected to a 6 h fast, after which they received an intraperitoneal (IP) injection of 1 g/kg glucose. Blood samples were collected from the tail vein at 0, 15, 30, 45, 60, and 120 min postinjection and analyzed using a handheld glucometer (ACCU‐CHEK active glucometer, Roche).

For insulin tolerance tests, mice underwent a 6 h fast and were subsequently injected with 0.55 U/kg of insulin. Blood samples were obtained from the tail vein at 0, 15, 30, 45, 60, and 90 min postinjection and analyzed using the Roche glucometer.

### 4.27 Statistical Analysis

Statistical analyses were conducted via the GraphPad Prism 8.0. Data are presented as mean ± standard deviation from at least three independent experiments for each cell type and animal group. Differences between groups were analyzed using Student's *t*‐tests. Statistical significance was defined as *p* < 0.05 (in all figures: *, *p* < 0.05; **, *p* < 0.01; ***, *p* < 0.001; **, *p* < 0.0001; NS = not significant).

## Author Contributions

W.Y.H. wrote the manuscript, designed and performed the most of the experiments, analyzed the data; X.Y.L., X.X.S., L.D.J., Q.Z., H.W.Z., L.L., and F.Q.W. performed the animal experiments; L.D.J., Y.M., Y.J.D., J.F.S., W.F.E.A.N., and H.Z. performed the in vitro experiments; L.M.X. performed the RNA‐seq, ATAC‐seq analysis; D.D.W. and W.Q.X., contributed to the experiments; W.L. provided human adipose tissue; H.W.J, J.L., Y.C., F.L., and W.H., were involved in discussing the data; T.D. conceived and supervised the study and revised the manuscript; all authors discussed and edited the manuscript.

## Funding

This work was supported by the Key Program of the National Natural Science Foundation of China (Grant No. 82130024), the National Key R&D Program of China (Grant No. 2023YFC3603404), the Fund for International Cooperation and Exchange of the National Natural Science Foundation of China (Grant No. 8231101033), Noncommunicable Chronic Diseases‐National Science and Technology Major Project (Grant No. 2025ZD0550100), and the Science and Technology Innovation Program of Hunan Province (Grant No. 2024RC3052).

## Conflicts of Interest

The authors declare no conflicts of interest.

## Supporting information




**Supporting File 1**: advs74754‐sup‐0001‐SuppMat.docx.

## Data Availability

The data supporting the findings of this study are available within the article and its Supporting Information. The RNA‐seq and ATAC‐seq data generated in this study have been deposited in the Gene Expression Omnibus (GEO) under accession number GSE274245 and GSE317566. The data that support the findings of this study are available in the supplementary material of this article.
