# Supplementary material for: Adhesion‐Related Macrophages Regulate Metabolic Homeostasis Through CAV‐1 Dependency
Source: Adv Sci (Weinh). 2026 Mar 12;13(26):e20936. doi: 10.1002/advs.202520936 (PMC13159151; doi:10.1002/advs.202520936)
Supplement: Supplementary file 1 — Supporting File 1: advs74754‐sup‐0001‐SuppMat.docx. [file ADVS-13-e20936-s001.docx]

Supporting Information

**Adhesion-Related Macrophages Regulate Metabolic Homeostasis through CAV-1 Dependency**

Wanyu Hu, Xiyan Liao, Limin Xi, Xiaoxiao Sun, Luodi Jing, Qin Zeng, Hongwei Jiang, Haowei Zhang, Lei Li, Fanqi Wang, Dandan Wang, Ying Mei, Yujin Din, Jianfeng Song, Wufuer Aini, Hui Zhou, Wanqin Xie, Helong Dai, Wei Liu, Joey Liu, Yan Cheng, Feng Liu, Willa A. Hsueh, and Tuo Deng*

**This file includes:**

Supplementary Figures. S1-18

Supplementary Tables. S1-2

**
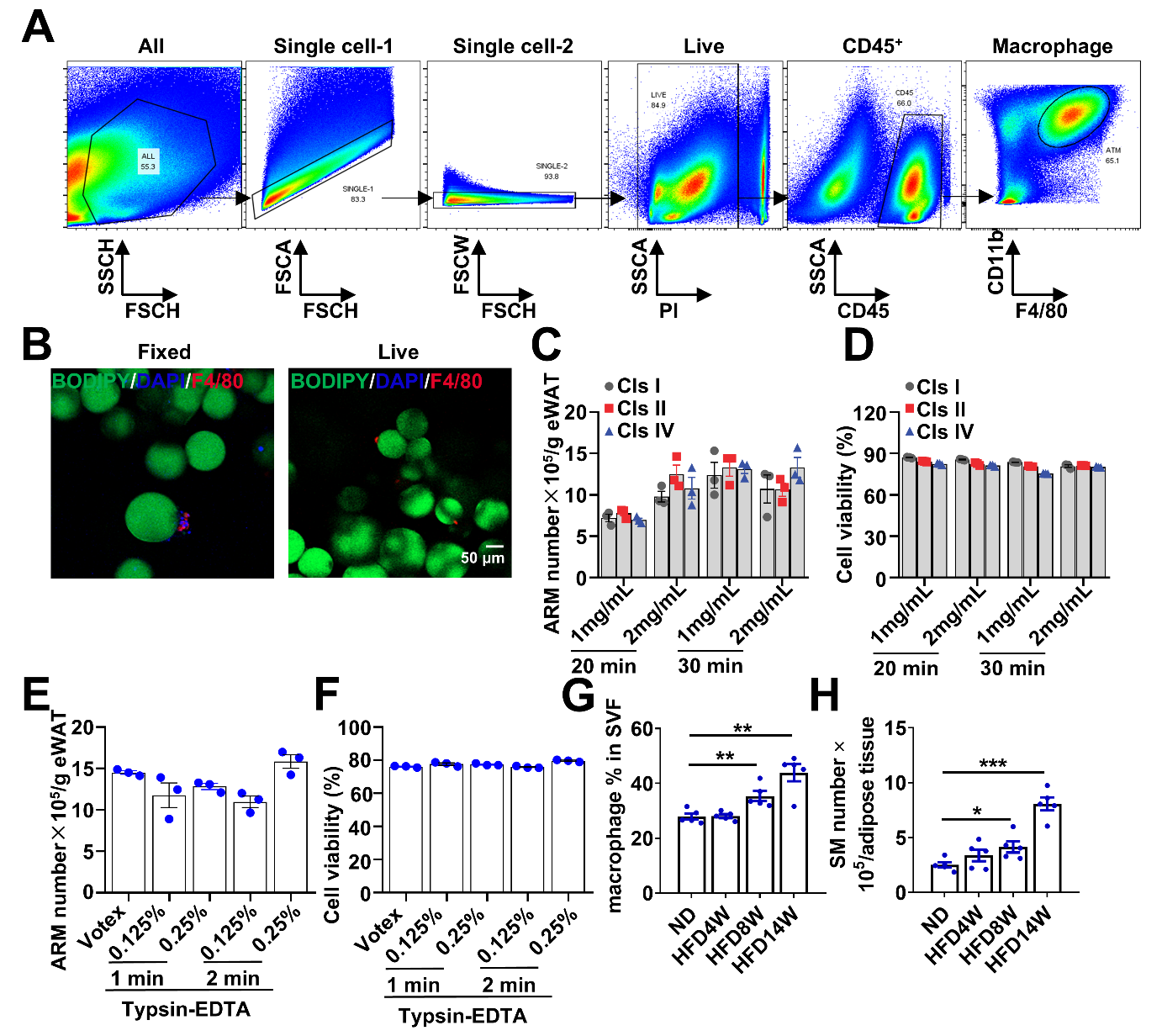
**

**Figure S1.** Isolation of ARMs in mice. (A) Gating strategy of FACS for ATM isolation from eWAT of mice. (B) Representative microscopy images showing ARMs adhered to adipocytes in eWAT under paraformaldehyde-fixed (left) and live-cell (right) conditions. Scale bar, 50 μm. (C–D) Flow cytometric analysis of (C) ARM abundance and (D) cell viability in eWAT from HFD-fed mice under different collagenase digestion conditions, (n = 3 per group). (E–F) Flow cytometric analysis of (E) ARM abundance and (F) cell viability in eWAT from HFD-fed mice after digestion with 1 mg/mL collagenase type II for 30 min, followed by trypsin treatment at different concentrations and incubation times, (n = 3 per group). (G-H) Quantitation of SMs subpopulations in epididymal fat pads. (G) Data are presented the percentage of SM in SVF of eWAT, (H) Data are presented as total number of macrophages per eWAT fat pad, (n = 5 per group). Data are expressed as means ± SEM. **p*< 0.05, ***p*< 0.01, ****p*< 0.001.


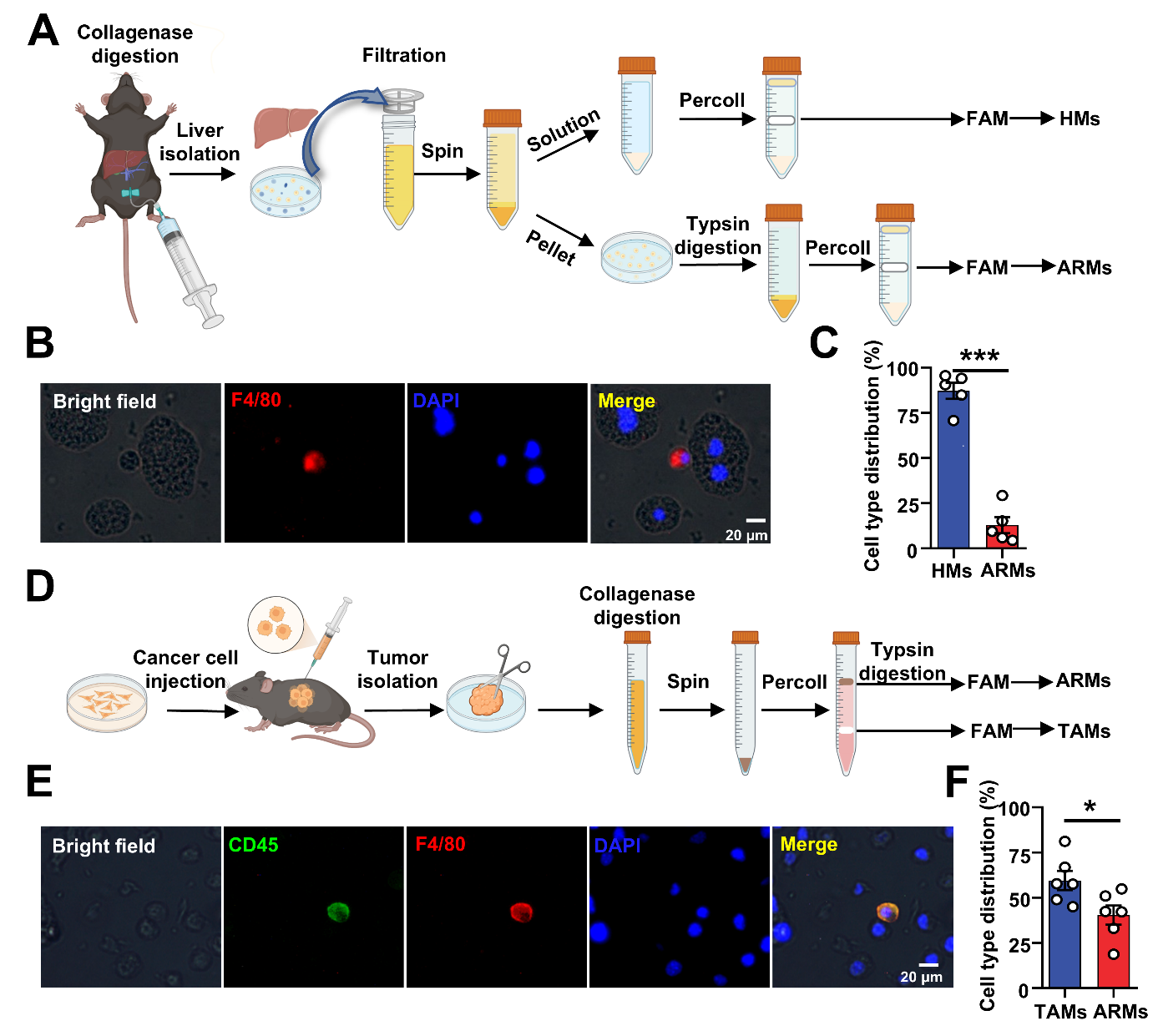


**Figure S2.** Conserved distribution of ARM in mice. (A) Schematic illustration depicting the isolation process of ARMs and hepatic macrophages (HMs) from the liver. The liver underwent in situ digestion and filtration. Subsequent centrifugation and washing separated hepatocytes from hepatic stromal cells. Hepatocytes were trypsin-digested, followed by Percoll isolation for single-nuclear cells, from which ARMs were isolated using FCM. Similarly, hepatic stromal cells went through Percoll and FCM to retrieve HMs. (B) Microscopy image of ARMs adhered to hepatocytes in liver of mice. Scale bar, 20 μm. (C) FCM analysis of ARMs and HMs distribution in liver of mice. n = 5 per group. (D) Schematic illustration depicting the isolation process of ARMs and tumor associated macrophages (TAMs) from the MC38 tumor-bearing mice. Tumor underwent digestion and filtration. Subsequent Percoll separation to obtain single nuclear cells and the tissue cell layer. Tissue cell layer was subjected to a 50-second trypsin digestion, from which ARMs were isolated using FCM. Similarly, single nuclear cells went through FCM to retrieve TAMs. (E) Microscopy image of ARMs adhered to tumor cells. Scale bar, 20 μm. (F) FCM analysis of ARMs and TAMs distribution in MC38 tumor-bearing mice. n = 6 per group. Data are expressed as means ± SEM. **p*< 0.05, ****p*< 0.001.


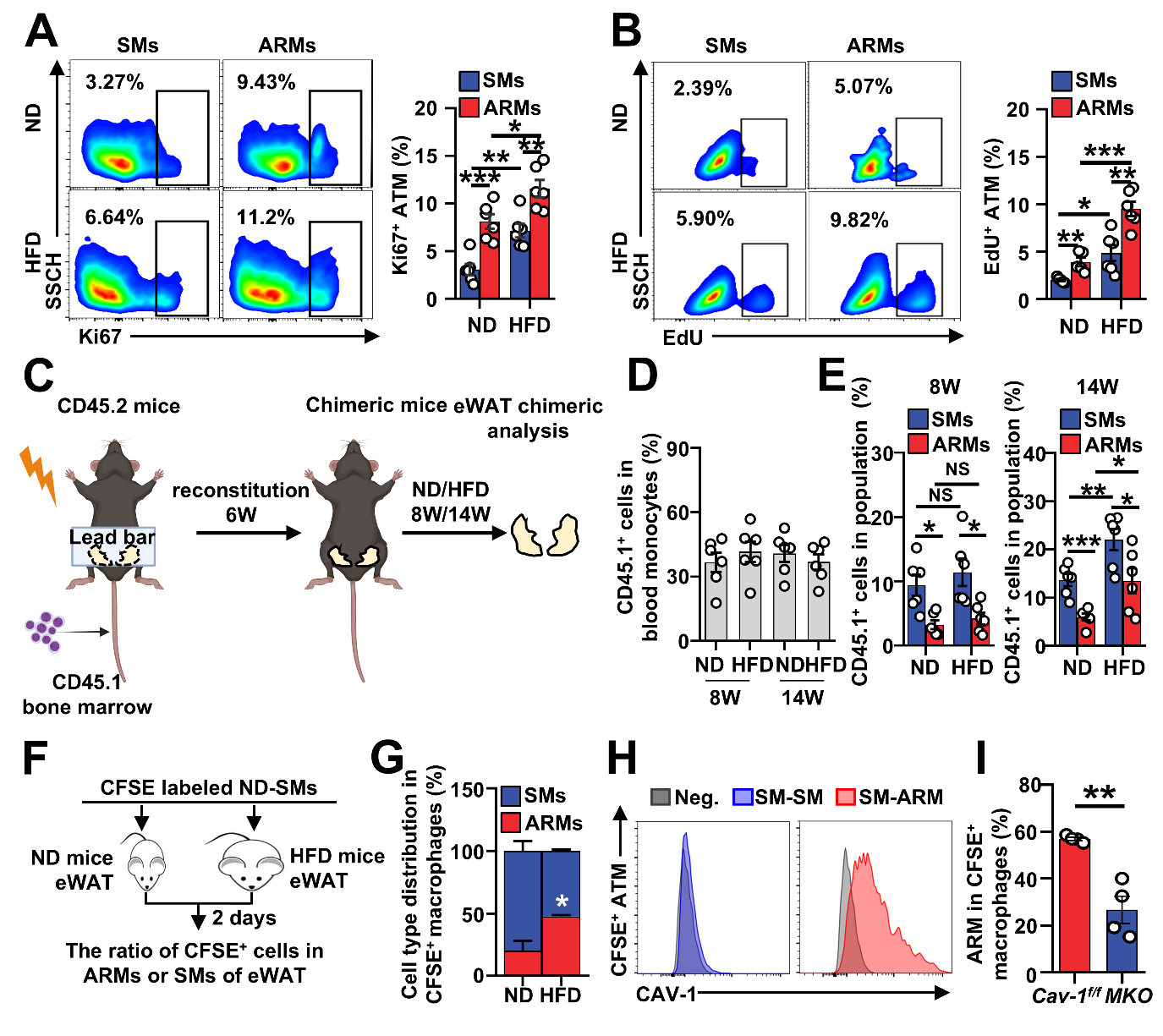


**Figure S3.** SM to ARM transformation and in situ proliferation drive ARM accumulation in obesity. (A-B) FCM analysis of (A) Ki67 expression and (B) EdU incorporation in ARMs and SMs from eWAT of ND and HFD mice. n ≥ 4 per group. (C) Schematic representation illustrating the eWAT-shielded irradiation and bone marrow transplantation procedure. CD45.2 mice were shielded with lead above the lower abdomen before receiving lethal irradiation. They were transplanted with CD45.1 bone marrow, allowing for reconstitution over 6 weeks, followed by 8-week or 14-week HFD. Chimerism analysis was conducted on eWAT. n = 6 per group. (D) Percentage of chimerism in blood monocytes (CD45^+^CD11b^+^CD115^+^). (E) Percentage of chimerism in SM and ARM. (F) Schematic description of the SMs shift assay. CFSE-labeled SMs from ND mice were injected into eWAT of ND or HFD mice. Two days post-operation, the proportion of ARMs and SMs among CFSE-labeled macrophages in the eWAT of transplanted mice was assessed. (G) Cell type distribution within CFSE-labeled macrophages. n = 4 per group. (H) FCM analysis of CAV-1 staining CAV-1 expression in CFSE⁺ ARMs and SMs (Neg., CFSE⁺ ATM FMO). (I) CFSE-labeled SMs isolated from ND-fed *Cav-1^f/f^* and *MKO* mice were injected into the eWAT of HFD-fed recipient mice. Two days after transplantation, the proportion of ARMs among CFSE-labeled macrophages in recipient mice was quantified, n = 4 per group. Data are expressed as means ± SEM. **p*< 0.05, ***p*< 0.01, ****p*< 0.001.

**
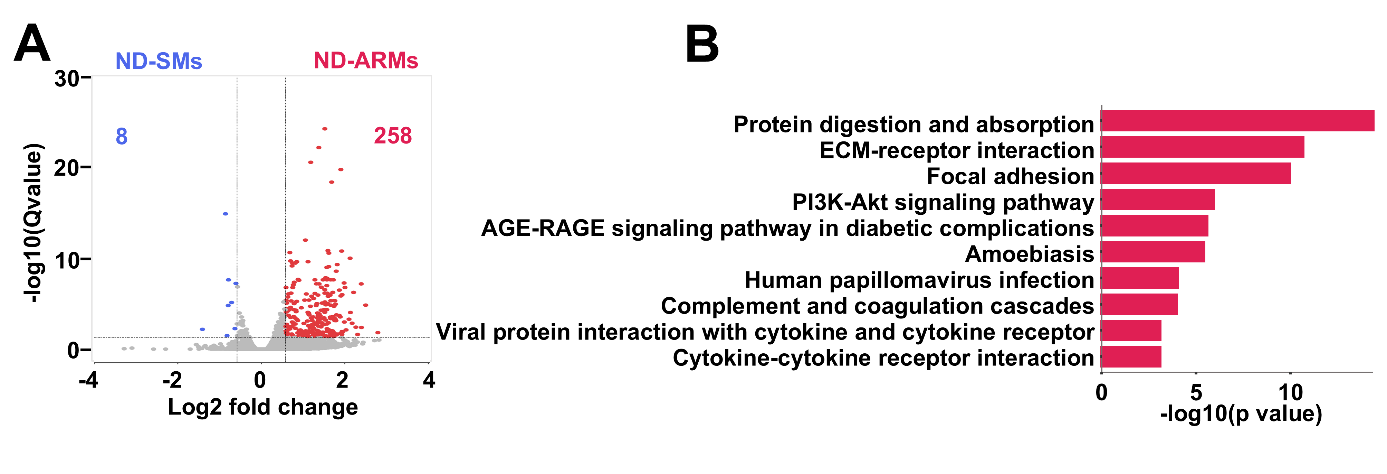
**

**Figure S4.** ARMs in ND mice exhibit a gene landscape for adhesion. (A) Volcano plot depicting differentially expressed genes between FACS-sorted ARMs and SMs from ND mice (fold change > 1.5, false discovery rate < 0.05, n = 2 per group, with 10 mice pooled per sample). (B) KEGG pathway analysis of ARMs upregulated gene, illustrating the top ten most significant pathways in ND mice.

**
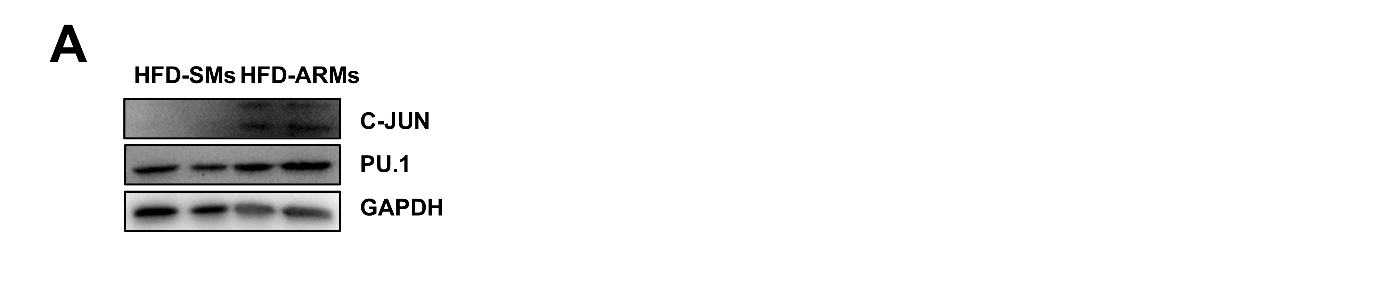
**

**Figure S5.** ARMs exhibit higher c-JUN expression. (A) Western blot analysis of c-JUN and PU.1 expression in ARMs and SMs from HFD-fed mice.

**
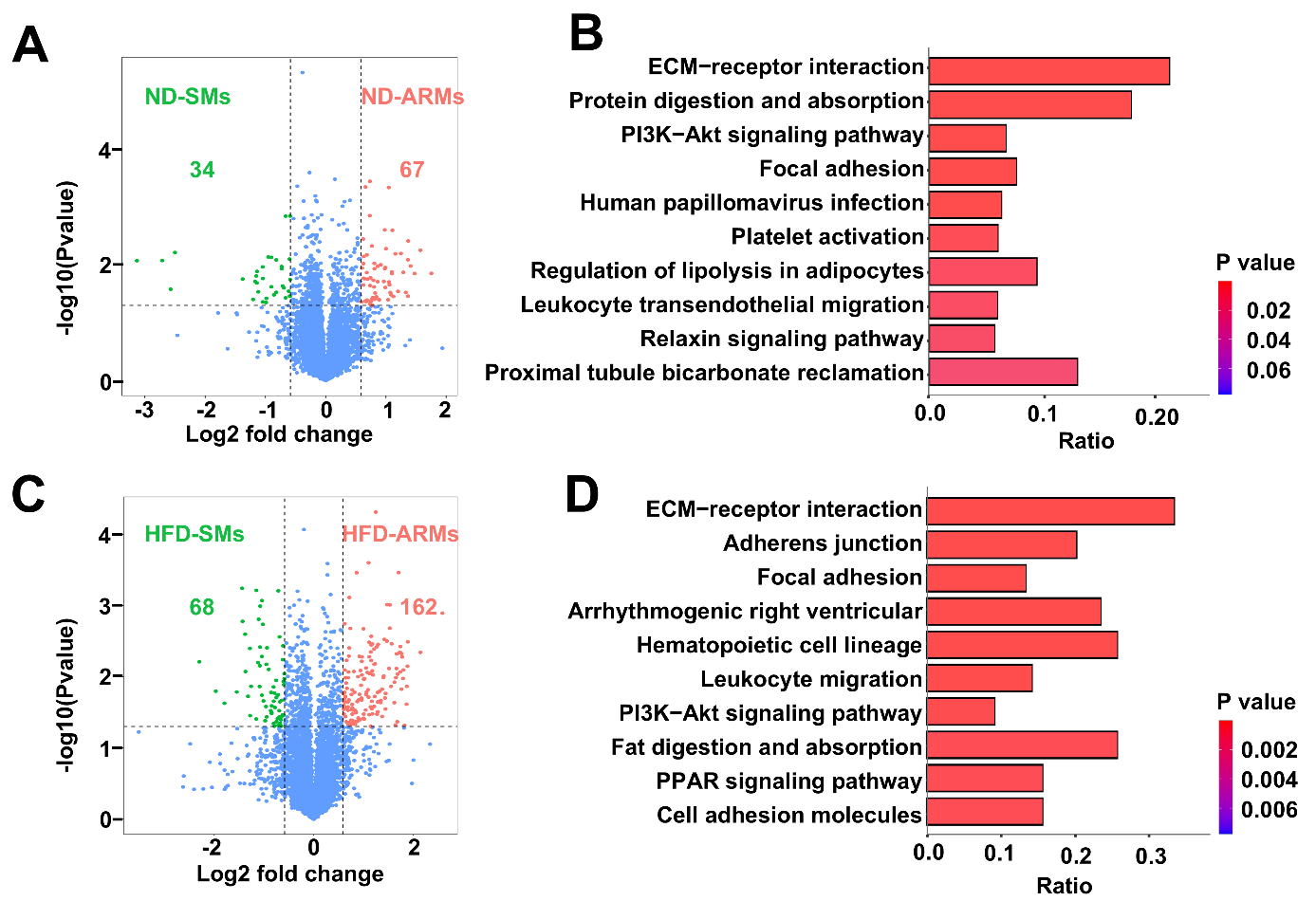
**

**Figure S6.** ARMs exhibit adhesion- and lipid-processing–related proteomic features. (A) Volcano plot showing differentially expressed proteins between FACS-sorted ARMs and SMs from ND-fed mice (fold change > 1.5, false discovery rate < 0.05; n = 3 per group, with 6 mice pooled per sample). (B) KEGG pathway analysis of proteins upregulated in ARMs, showing the top ten most significantly enriched pathways in ND-fed mice. (C) Volcano plot showing differentially expressed proteins between FACS-sorted ARMs and SMs from HFD-fed mice (fold change > 1.5, false discovery rate < 0.05; n = 3 per group, with 4 mice pooled per sample). (D) KEGG pathway analysis of proteins upregulated in ARMs, showing the top ten most significantly enriched pathways in HFD-fed mice.


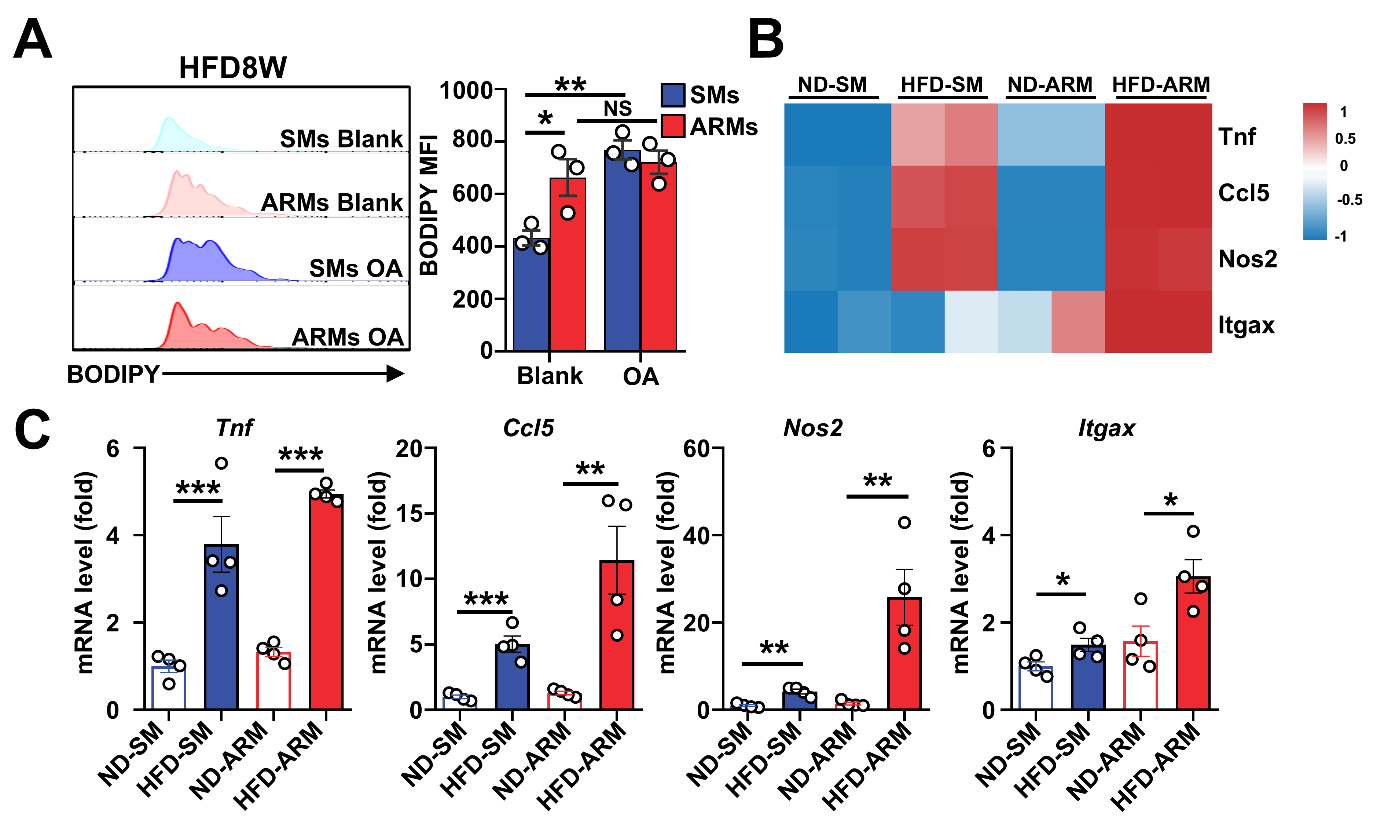


**Figure S7.** Prolonged HFD exposure exceeds the functional capacity of ARMs, inducing a low-grade pro-inflammatory phenotype. (A) FCM analysis of BODIPY staining in ARMs and SMs from HFD 8W mice after lipid-loaded with oleate acid (OA) for 4 h or BSA (Blank), and quantification of BODIPY mean fluorescence intensity (MFI) levels (n = 3). (B) Heatmap displaying the expression of inflammation genes in ARMs and SMs between ND mice and HFD mice. (C) Gene expression profiles of inflammation in ARMs and SMs isolated from ND mice and HFD 8W mice (n =4 per group, with 3 mice pooled per sample). Data are expressed as means ± SEM. **p*< 0.05, ***p*< 0.01, ****p*< 0.001.


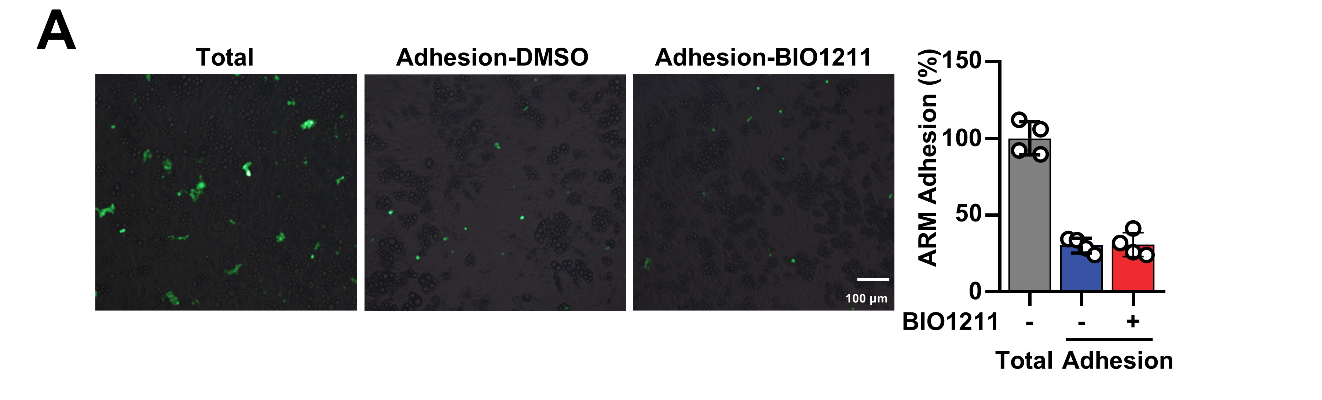


**Figure S8.** Adhesion of ARMs to adipocytes is independent of α4 integrin. (A) Adipocyte attachment assay. ARMs isolated from HFD-fed mice were bead-purified, CFSE-labeled, and seeded onto adipocytes that were either pretreated or not pretreated with 5 nM BIO1211 (α4 integrin inhibitor) for 16 h. After 30 min of incubation, non-adherent cells were washed away, and the remaining adherent ARMs were fixed and quantified (n = 3). Scale bar, 100 μm. Data are expressed as means ± SEM.

**
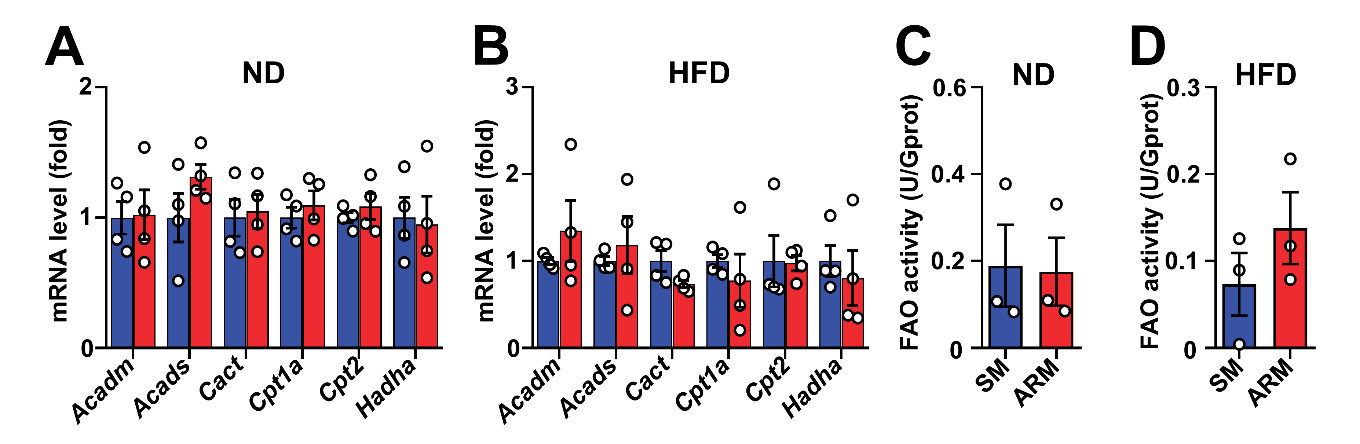
**

**Figure S9.** No significant difference in fatty acid oxidation between ARMs and SMs. (A) Gene expression profiles of fatty acid β-oxidation genes in ARMs and SMs isolated from ND-fed mice (n = 4 per group, with 3 mice pooled per sample). (B) Gene expression profiles of fatty acid β-oxidation genes in ARMs and SMs isolated from HFD-fed mice (n = 4 per group, with 3 mice pooled per sample). (C–D) Fatty acid oxidation assays in ARMs and SMs isolated from (C) ND-fed or (D) HFD-fed mice (n = 3 per group, with 6 mice pooled per sample). Data are expressed as means ± SEM.

**
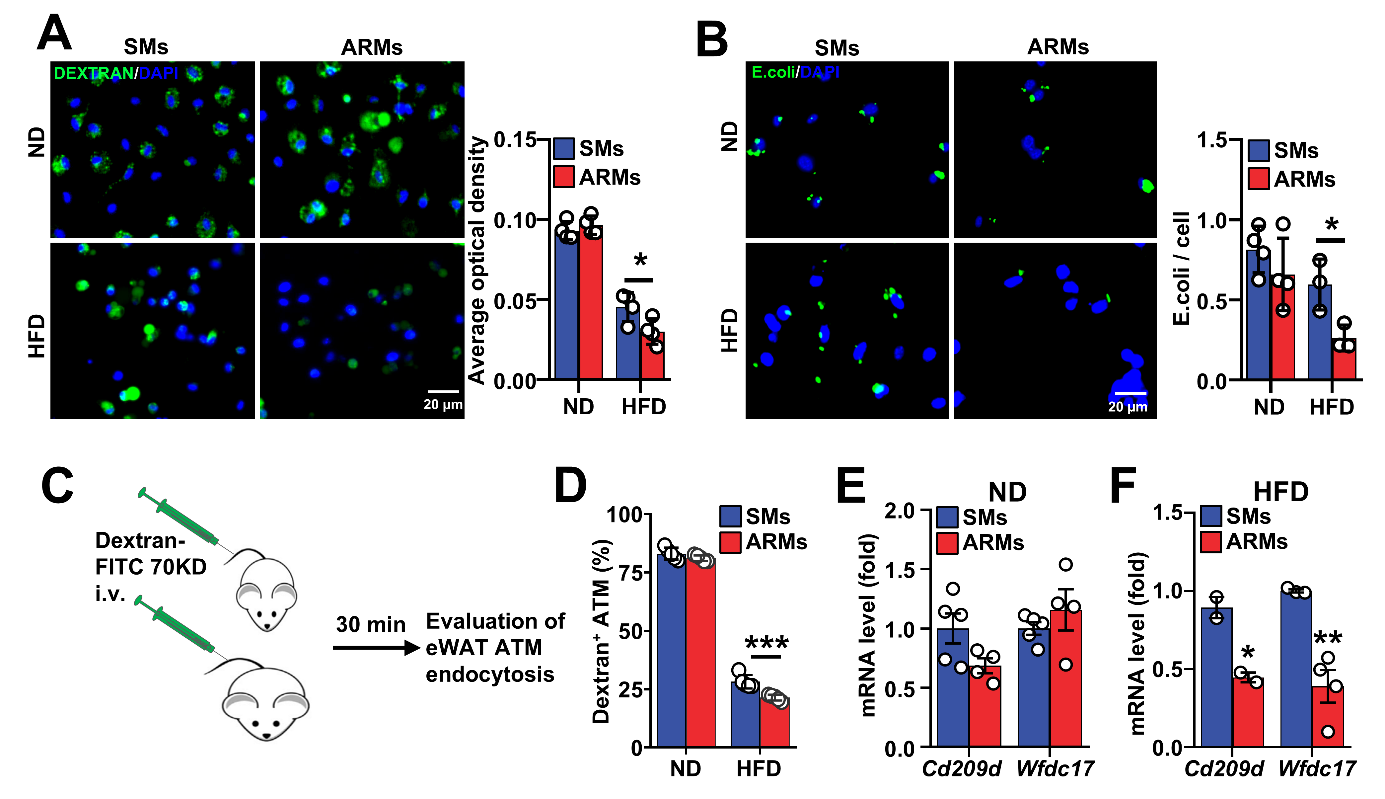
**

**Figure S10.** ARMs display attenuated engulfment capability. (A) Microscopy analysis of dextran-FITC uptake for 30 min in ARMs and SMs from both ND and HFD 8W mice. Scale bar, 20 μm. (B) Microscopy analysis of E. coli-CFSE phagocytosis for 30 min in ARMs and SMs from ND and HFD 8W mice. Scale bar, 20 μm. (C-D) In vivo endocytosis assay. (C) dextran-FITC 70 kD was injected i.v. 30 min after the injection, ATMs’ endocytic activity was evaluated by FCM. (D) Percentage of dextran^+^ macrophages among ARMs and SMs from ND and HFD 8W mice (n = 5). (E-F) Gene expression of phagocytosis in ARMs and SMs isolated from (E) ND mice and (F) HFD 8W mice (n =2-5 per group, with 6 mice pooled per sample). Data are expressed as means ± SEM. **p*< 0.05, ***p*< 0.01, ****p*< 0.001.

**
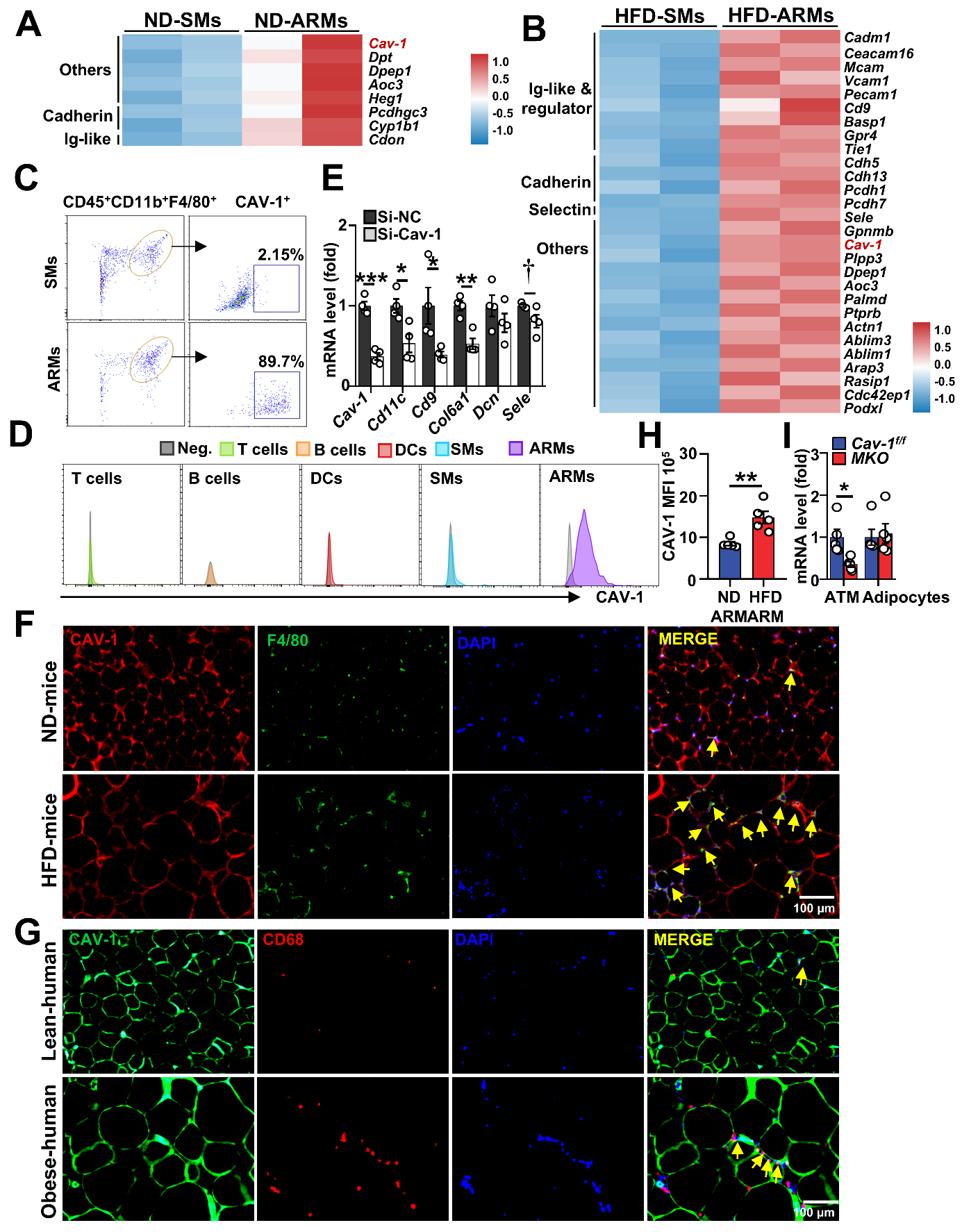
**

**Figure S11.** CAV-1 as a marker of ARMs. (A-B) Heatmap of adhesion genes differentially expressed between SMs and ARMs in (A) ND mice and (B) HFD mice. (C) FCM plots showing the gating strategy for CAV-1 expression in ATMs (CD45^+^CD11b^+^F4/80^+^) from HFD 6W mice. (D) CAV-1 expression in major immune cell types in the SVF of HFD 6W mice. (E) Effect of *Cav-1* siRNA on mRNA expression of *Cd11c*, *Cd9*, *Col6a1*, *Dcn* and *Sele* in ARMs from HFD 8W mice (n = 4). (F) Representative immunofluorescence images of CAV-1 (red), F4/80 (green) and nuclei (blue) in eWAT from ND and HFD 8W mice. Scale bar, 100 μm. (G) Representative immunofluorescence images of CAV-1 (green), CD68 (red) and nuclei (blue) in VAT from lean and obese individuals. Scale bar, 100 μm. (H) MFI of CAV-1 in ARMs isolated from ND- and HFD-fed mice (n = 5 per group). (I) mRNA expression of *Cav-1* in ATMs and adipocytes isolated from eWAT of *Cav-1^f/f^* and *MKO* mice (n = 5 per group). Data are expressed as means ± SEM. **p*< 0.05, ***p*< 0.01, ****p*< 0.001.

**
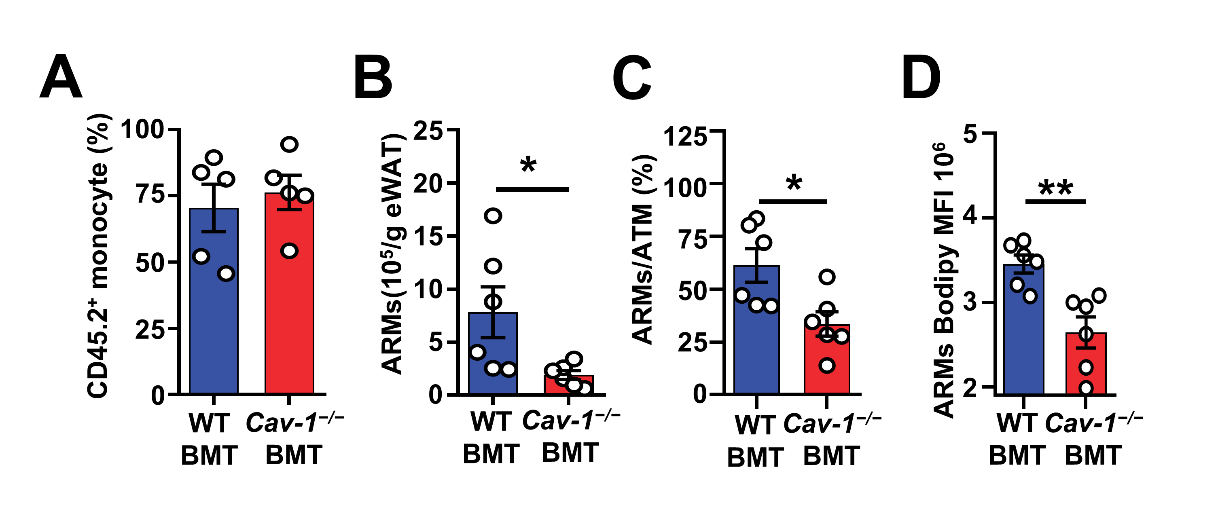
**

**Figure S12.** *Cav-1^−/−^* BMT reduces the number of ARMs and the intracellular lipid content. (A) Percentage of reconstitution in blood monocytes (CD45^+^CD11b^+^CD115^+^) post bone marrow transplantation from CD45.2 donors (*B6* or *Cav-1^−/−^*) into CD45.1 recipient mice (*B6*). (B-C) FCM analysis of ARMs abundance (B), and ARMs distribution (C) within ATM compartment in eWAT from WT BMT and *Cav-1^−/−^* BMT mice after 10W HFD (n = 6 per group). (D) FCM analysis of BODIPY staining in ARMs from eWAT of WT BMT and *Cav-1^−/−^* BMT mice after 10W HFD (n = 6 per group). Data are expressed as means ± SEM. **p*< 0.05, ***p*< 0.01.

**
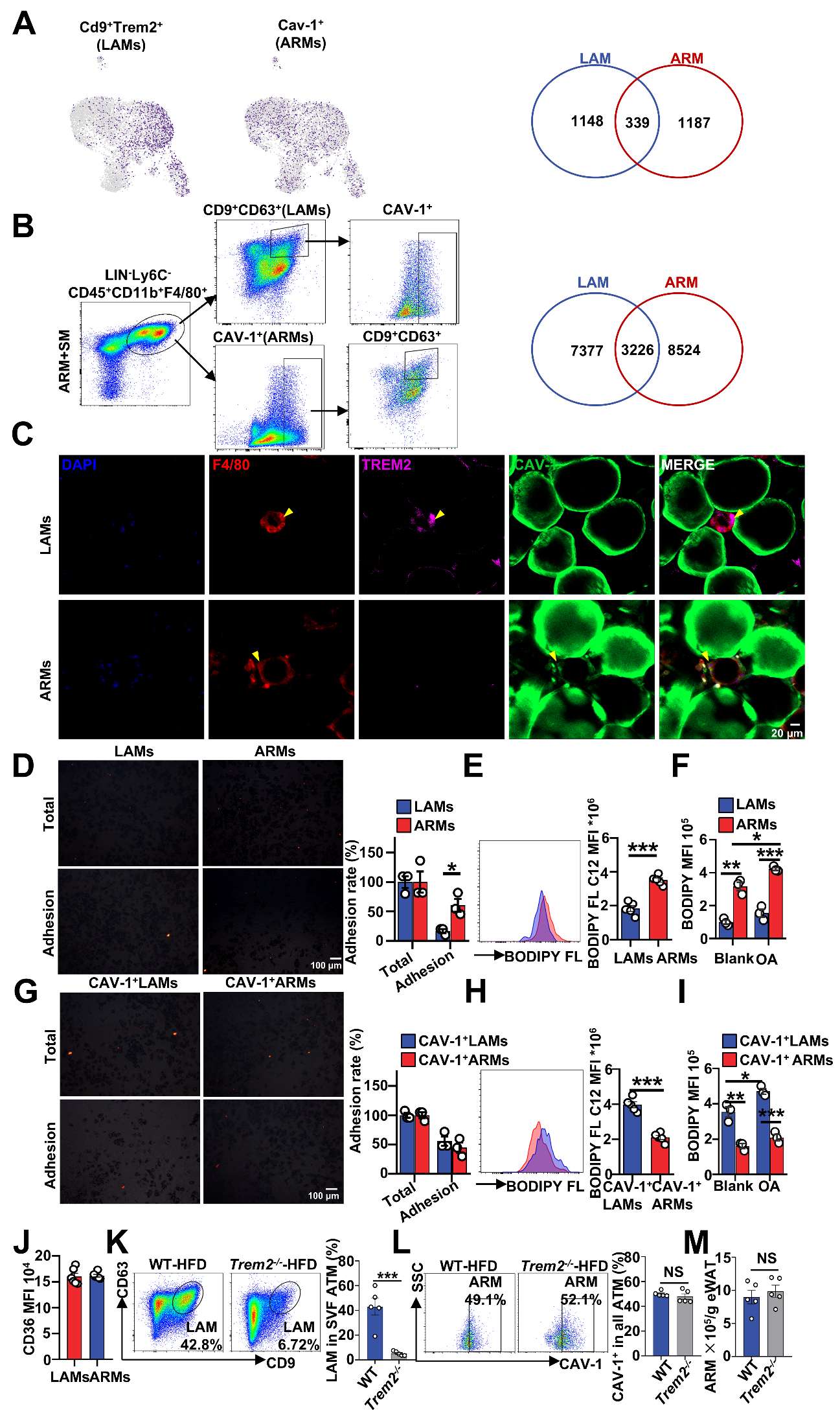
**

**Figure S13.** ARMs Are Distinct from LAMs. (A) UMAP of LAMs and ARMs in single-nucleus RNA sequencing of eWAT from mice, statistical overlap between LAMs and ARMs. (B) Gating strategy of FACS for LAM and ARM isolation from eWAT of mice, statistical overlap between LAMs and ARMs. (C) Representative immunofluorescence images of CAV-1 (green), TREM2 (purple), F4/80 (red) and nuclei (blue) in eWAT from HFD 8W mice. Scale bar, 100 μm. (D) Adipocyte attachment assay. ARMs and LAMS isolated from HFD-fed mice were seeded onto adipocytes After 30 min of incubation, non-adherent cells were washed away, and the remaining adherent ARMs were fixed and quantified (n = 3). Scale bar, 100 μm. (E) Fatty acid trafficking in ARMs and LAMs isolated from HFD 10W mice loaded with BODIPY FL C12 for 30 min. (F) FCM analysis of BODIPY staining in ARMs and SMs from HFD 10W mice after lipid-loaded with oleate acid (OA) for 4 h or BSA (Blank). (G) Adipocyte attachment assay. CAV-1+LAMs (CD9+CD63+CAV-1+) and CAV-1+ARMs (CD9-CD63-CAV-1+) isolated from HFD-fed mice were seeded onto adipocytes After 30 min of incubation, non-adherent cells were washed away, and the remaining adherent ARMs were fixed and quantified (n = 3). Scale bar, 100 μm. (H) Fatty acid trafficking in CAV-1+LAMs(CD9+CD63+CAV-1+) and CAV-1+ARMs (CD9-CD63-CAV-1+) isolated from HFD 10W mice loaded with BODIPY FL C12 for 30 min. (I) FCM analysis of BODIPY staining in CAV-1+LAMs(CD9+CD63+CAV-1+) and CAV-1+ARMs (CD9-CD63-CAV-1+) from HFD 10W mice after lipid-loaded with oleate acid (OA) for 4 h or BSA (Blank). (J) FCM analysis of CD36 in ARMs and LAMs of HFD mice (n = 6 per group). (K-L) FCM analysis of (K)LAMs and (L) ARMs in WT and *Trem2^−/−^* mice after 10W HFD (n = 5). (M) FCM analysis of ARMs abundance in eWAT from in WT and *Trem2^−/−^* mice after 10W HFD (n = 5). Data are expressed as means ± SEM. **p*< 0.05, ***p*< 0.01, ****p*< 0.001, NS = not significant.

**
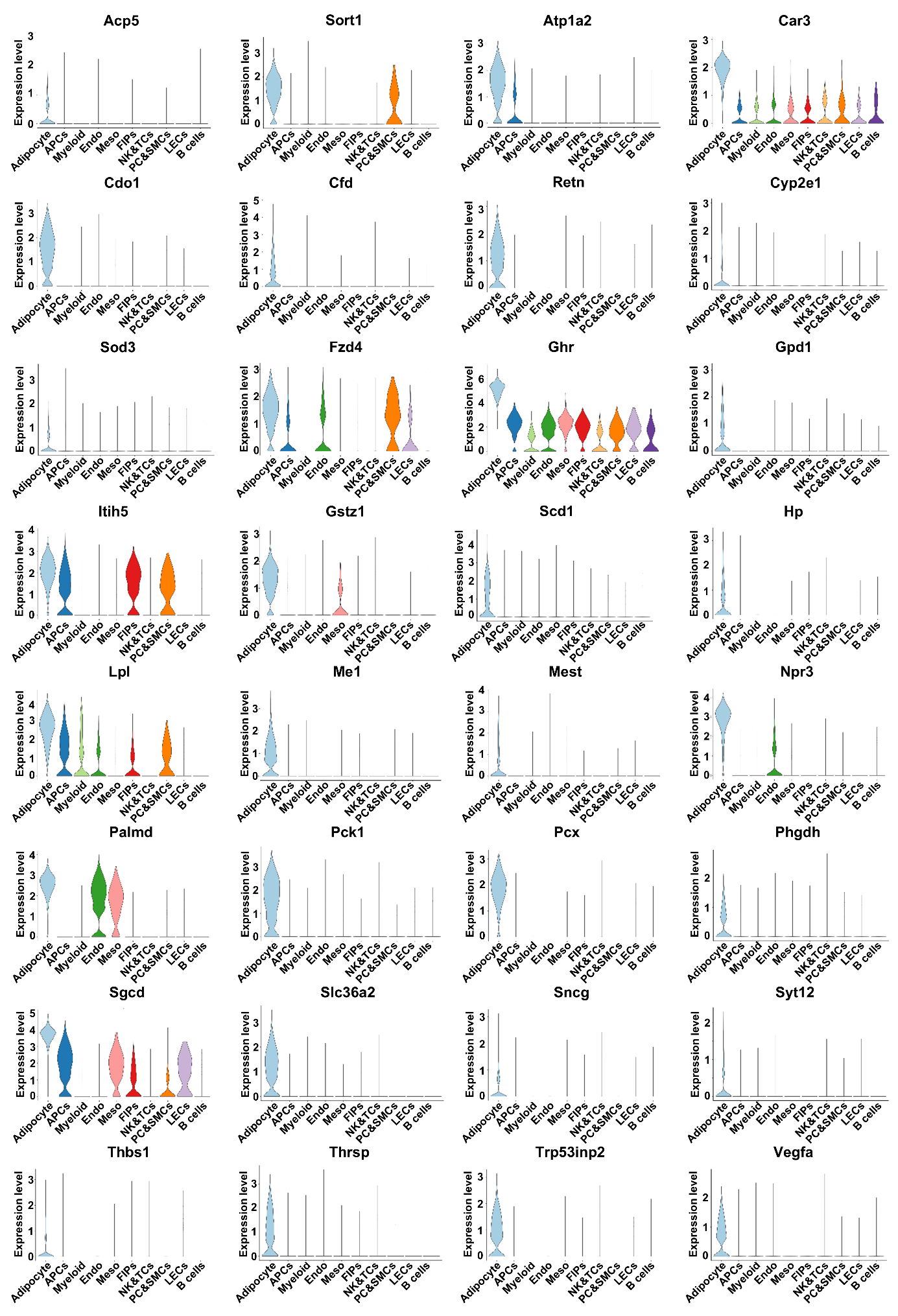
**

**Figure S14.** Violin plots of foreign ARM gene expression in different cell types from single nuclear sequencing data of eWAT.

**
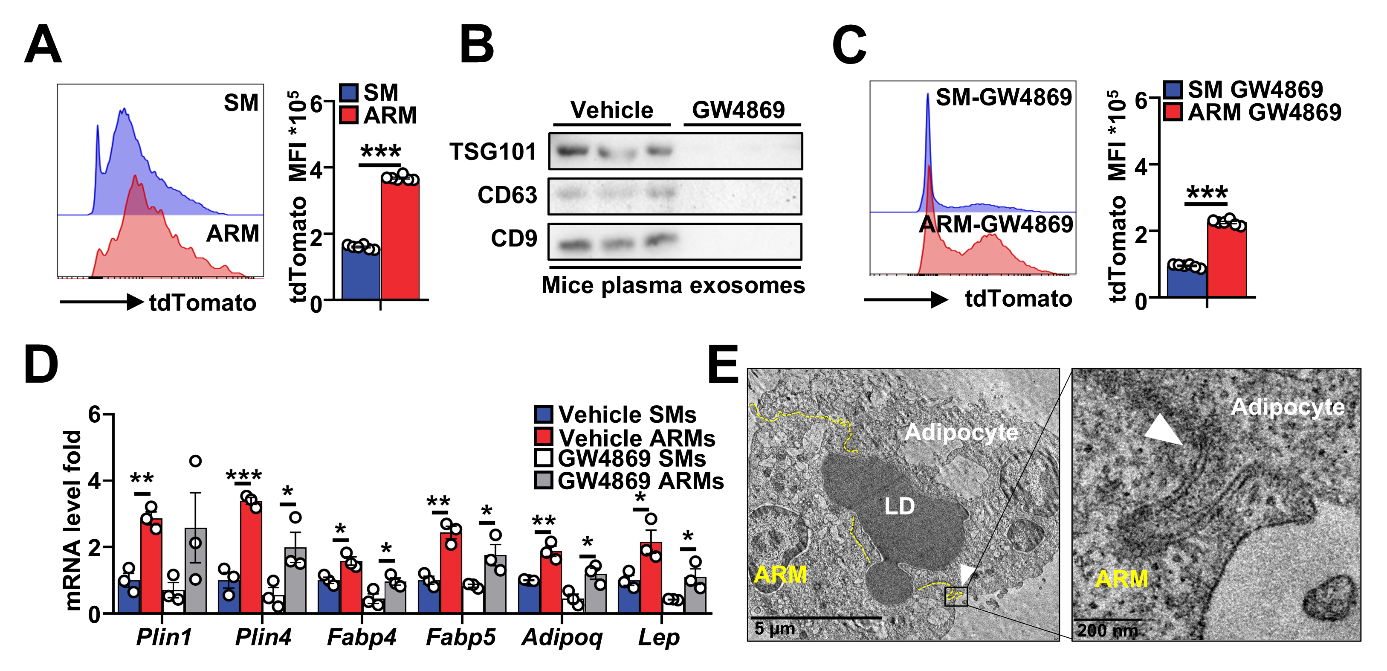
**

**Figure S15.** Exosomes did not affect the transfer of adipocyte RNA to ARMs. (A) FCM analysis of MFI of TdTomato in ARMs or SMs, n = 6 per group. (B) Western blot of exosome marker proteins in released exosomes isolated from mice plasma. (C) FCM analysis of the MFI of TdTomato in ARMs or SMs after 1 week of GW4869 treatment, n = 6 per group. (D) The expression levels of foreign genes in ARMs after GW4869 treatment, n = 3 per group, with 2 mice pooled per sample. (E) Representative electron micrographs of the cell–cell contact interface between bead-purified ARMs and their adhered adipocytes isolated from 8-week HFD-fed mice. Scale bars: 5 μm and 200 nm. Data are expressed as means ± SEM. **p*< 0.05, ***p*< 0.01, ****p*< 0.001.

**
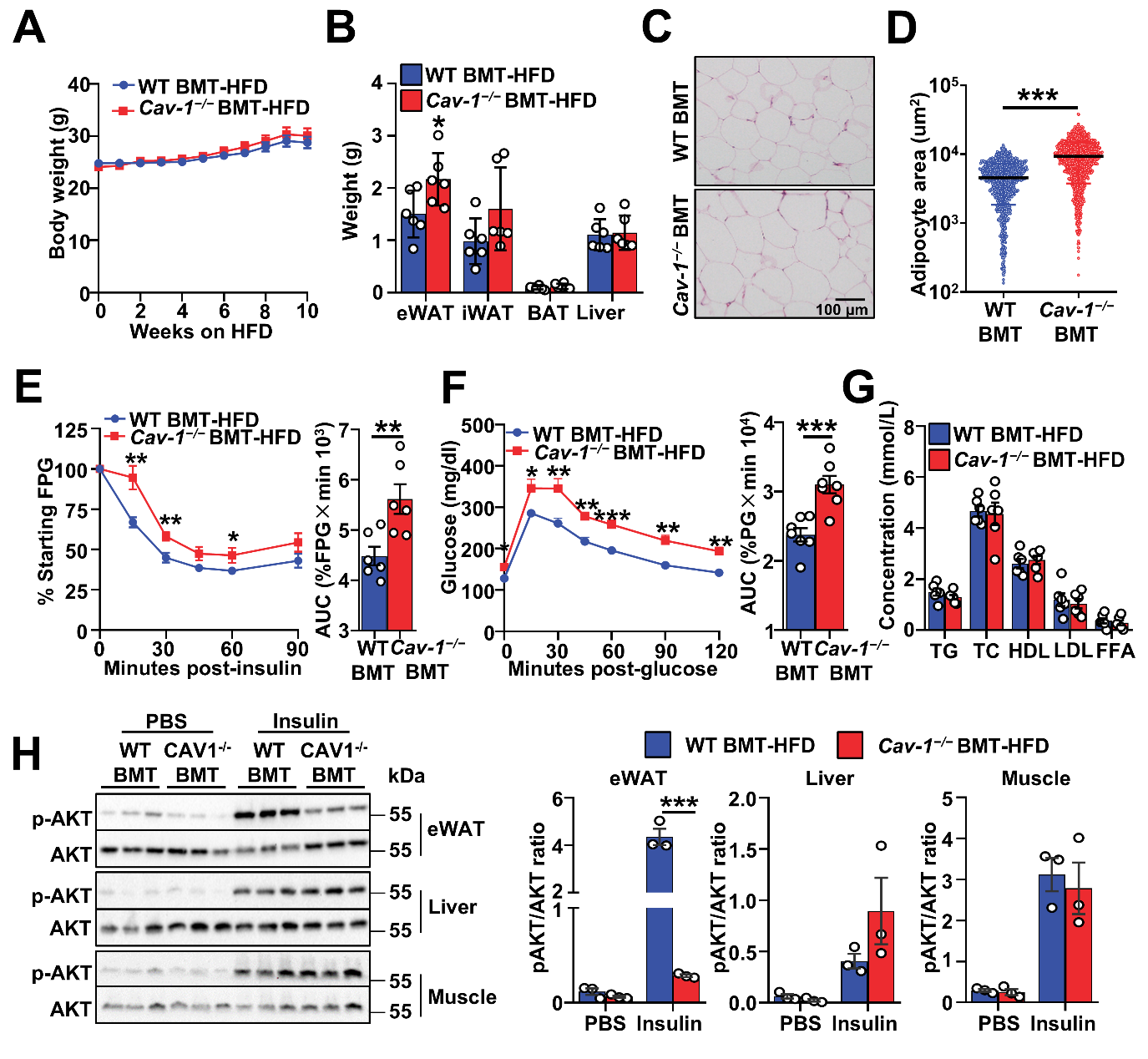
**

**Figure S16.** Immuno-cellular *Cav-1* prevents adipocyte hypertrophy and glycol-metabolic disorder. (A) Body weight over time on HFD, n = 6 per group. (B) Tissue weight at week 10 on HFD, n = 6 per group. (C) Images of H&E stain of eWAT sections. Scale bar, 100 μm.

(D) Quantification of the area of 1000 randomly selected adipocytes per genotype in H&E sections. (E) ITT in mice during week 8 of HFD feeding, n = 6 per group. (F) GTT in mice during week 10 of HFD feeding, n = 6 per group. (G) Triglyceride (TC), total cholesterol (TG), HDL, LDL, FFA (free fatty acids) levels in serum of mice during week 10 of HFD feeding. (n = 5-7 per group). (H) Immunoblots of lysates from eWAT, liver, and skeletal muscle with anti-pAKT and anti-AKT and quantitation of phosphorylated AKT normalized to total AKT. Data are expressed as means ± SEM. **p*< 0.05, ***p*< 0.01, ****p*< 0.001.

**
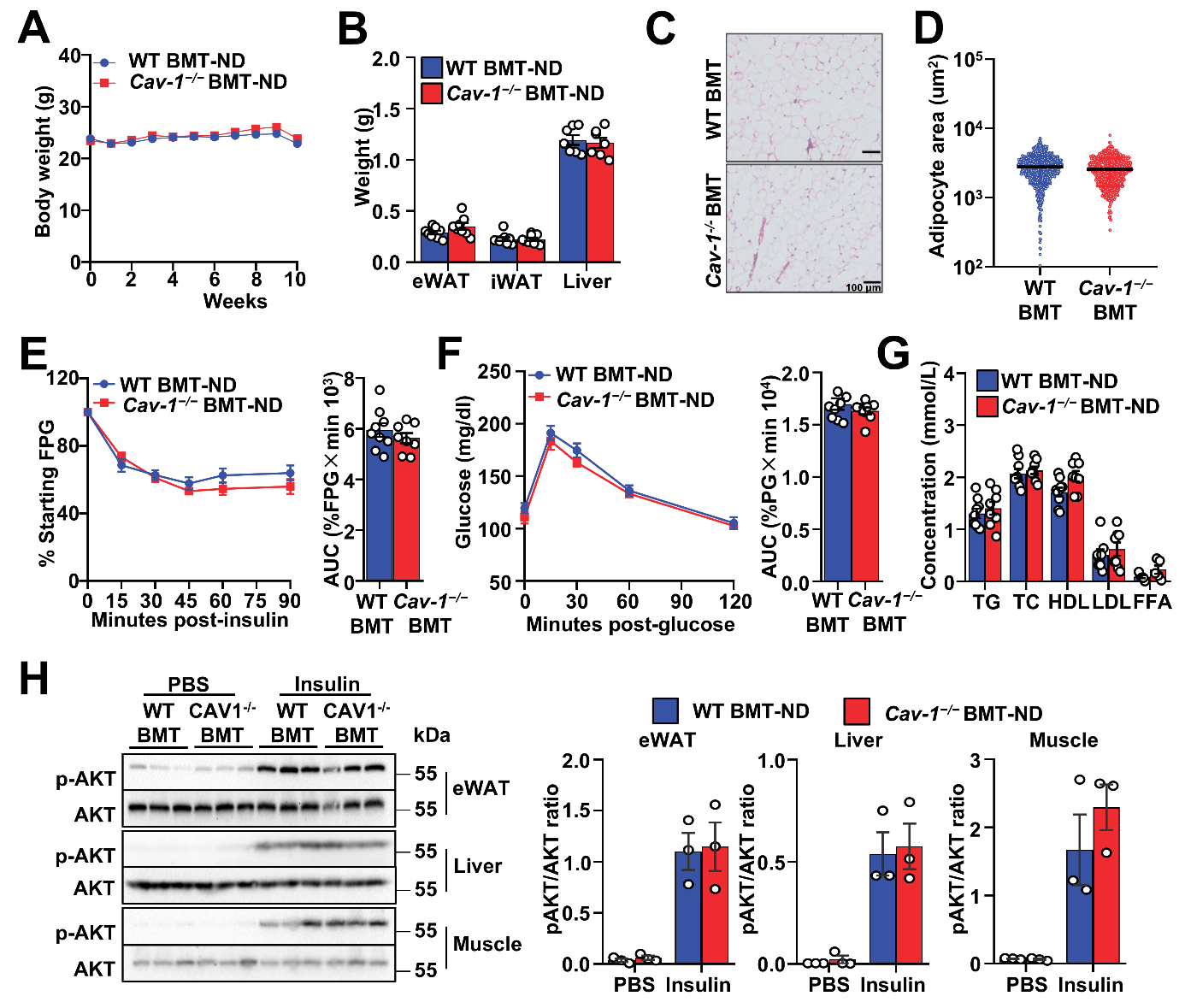
**

**Figure S17.** Immuno-cellular *Cav-1* exerts no effect on the metabolic phenotype of ND mice. (A) Body weight of chimeric mice over time on ND, n = 8-9 per group. (B) Tissue weight at week 10 on ND, n = 8-9 per group. (C) Images of H&E stain of eWAT sections. Scale bar, 100 μm. (D) Quantification of the area of 1000 randomly selected adipocytes per genotype in H&E sections. (E) ITT in mice during week 8 of ND feeding, n = 8-9 per group. (F) GTT in mice during week 10 of ND feeding, n = 8-9 per group. (G) Triglyceride (TC), total cholesterol (TG), HDL, LDL, FFA (free fatty acids) levels in serum of mice during week 10 of ND feeding. (n = 8-9 per group). (H) Immunoblots of lysates from eWAT, liver, and skeletal muscle with anti-pAKT and anti-AKT and quantitation of phosphorylated AKT normalized to total AKT. Data are expressed as means ± SEM.

**
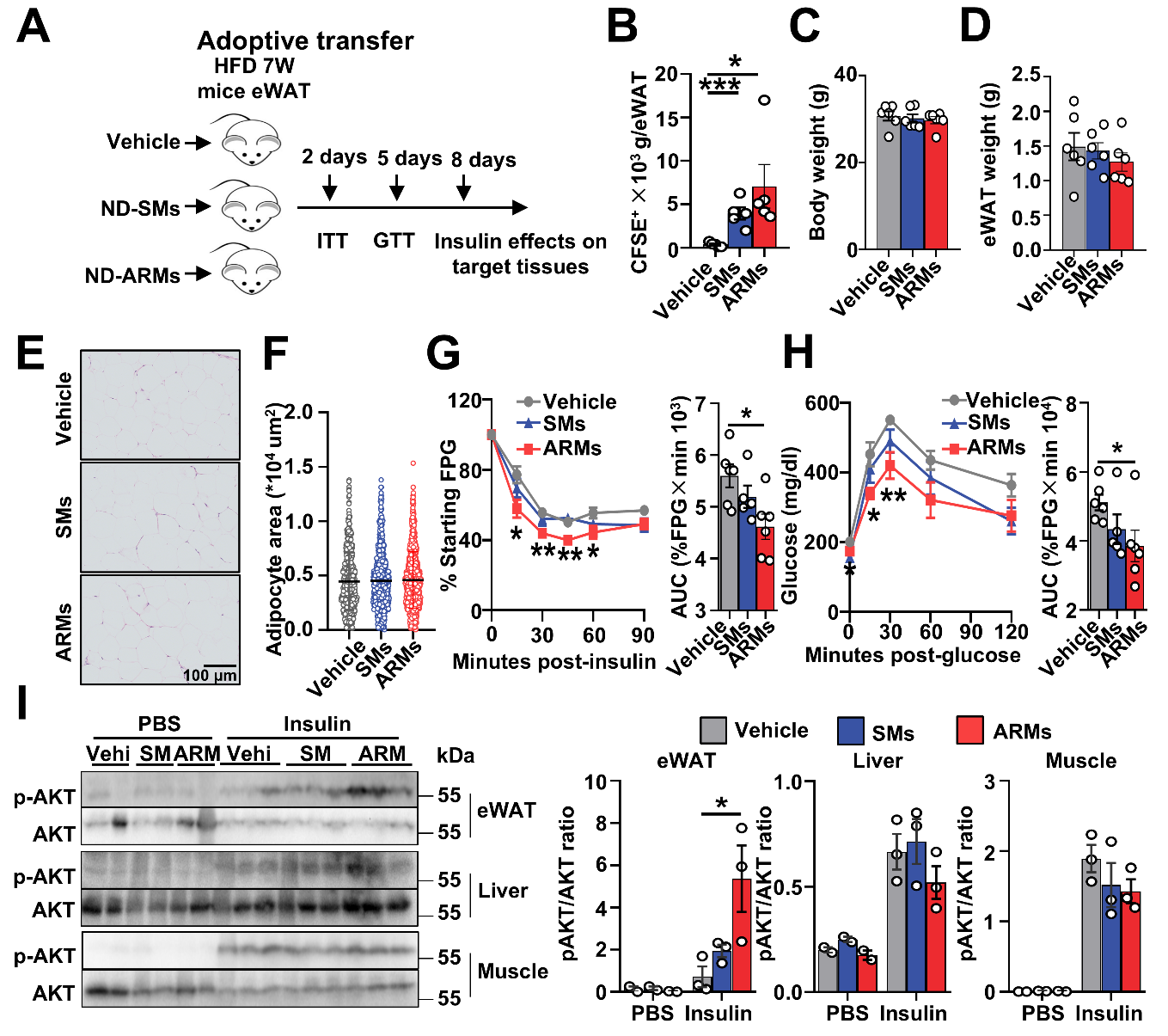
**

**Figure S18.** ND-ARMs alleviate obesity-induced insulin resistance. (A) Schematic depiction illustrating ARMs and SMs shift assay. ARMs and SMs from ND mice were resuspended in DMEM mixed with Matrigel and injected into eWAT of mice in the 7^th^ week of HFD. (B) Number of CFSE^+^ donor ARMs and SMs in recipient eWAT (n=6 per group). (C) Body weight of mice post-operation. (D) eWAT weight of mice post-operation. (E) Images of H&E stain of eWAT sections. Scale bar, 100 μm. (F) Quantification of the area of 500 randomly selected adipocytes per group in H&E sections. (G) ITT in mice post-operation. (H) GTT in mice post-operation. (I) Immunoblots of lysates from eWAT, liver, and skeletal muscle with anti-pAKT and anti-AKT and quantitation of phosphorylated AKT normalized to total AKT. Data are expressed as means ± SEM. Data are expressed as means ± SEM. **p*< 0.05, ****p*< 0.01, ****p*< 0.001.

**Table S1.** KEY RESOURCES TABLE

| REAGENT or RESOURCE | SOURCE | IDENTIFIER |
| --- | --- | --- |
| Antibodies |  |  |
| CD45 APC | BioLegend | Cat# 103112 |
| CD45 FITC | BioLegend | Cat#103108 |
| CD11b PerCP/Cyanine5.5 | BioLegend | Cat#101228 |
| F4/80 PE/Cyanine7 | BioLegend | Cat#123114 |
| F4/80 PE-DAZZLE594 | BioLegend | Cat#123146 |
| CD115 PE/Cyanine7 | BioLegend | Cat#135524 |
| Ki67 Alexa Fluor®700 | BioLegend | Cat#350530 |
| CD49a PE | BioLegend | Cat#142603 |
| CD9 PE | BioLegend | Cat#1224806 |
| CD3 APC | Biolegend | Cat#100236 |
| CD19 Alexa Fluor®700 | Biolegend | Cat#115528 |
| MHCII eFlour 450 | eBioscience | Cat#1995328 |
| CD144 APC | BioLegend | Cat#138011 |
| CD63 APC | BioLegend | Cat#143905 |
| CD36 APC | BioLegend | Cat#102611 |
| CD45.1 PerCP/Cyanine5.5 | BioLegend | Cat#110728 |
| CD45.2 APC/Cyanine7 | BioLegend | Cat#109824 |
| Anti- CAV-1 antibody | Cell Signaling Technology | Cat#D46G3 |
| Anti- AP-1 antibody | Cell Signaling Technology | Cat #9165T |
| Anti- PU.1 antibody | Cell Signaling Technology | Cat #2258T |
| Anti- TREM2 antibody | Cell Signaling Technology | Cat #55739T |
| Anti- CD68 antibody | Cell Signaling Technology | Cat#D489C |
| Anti- F4/80 antibody | Abcam | Cat#AB6640 |
| Anti-SR-BI/SCARB1 Antibody | BOSTER | Cat#A01093-1 |
| Anti-Perilipin 1 Antibody | Proteintech | Cat#27716-1-AP |
| Anti-pAKT antibody | Cell Signaling Technology | Cat#4060S |
| Anti-AKT antibody | Cell Signaling Technology | Cat#4691L |
| Goat anti-Rabbit IgG Secondary Antibody, Alexa Fluor™ 594 | Invitrogen | Cat#A-11012 |
| Goat anti-Rabbit IgG Secondary Antibody, Alexa Fluor™ 488 | Invitrogen | Cat#A-11034 |
| Goat anti-Rabbit IgG Secondary Antibody, Alexa Fluor™ 633 | Invitrogen | Cat#A-21070 |
| Goat anti-Rat IgG Secondary Antibody, Alexa Fluor™ 488 | Abcam | Cat#ab150157 |
| Opal 4-color Manual IHC Kit | PerkinElmer | Cat#2494755 |
| Chemicals, peptides, and recombinant proteins | | |
| FITC-dextran | Sigma-Aldrich | Cat#46945 |
| Zombie NIR | Biolegend | Cat#423106 |
| PI | Biolegend | Cat#421301 |
| BODIPY | Invitrogen | Cat#D3922 |
| BODIPY FL. C12 | Invitrogen | Cat#D3822 |
| DAPI | Cell Signaling Technology | Cat#4083 |
| HOCHEST 33342 | Cell Signaling Technology | Cat#4082 |
| Collagenase, Type II | Worthington Biochemical | Cat#LS004177 |
| Collagenase, Type IV | Worthington Biochemical | Cat#LS004189 |
| DNase I | Solarbio | Cat#D8070 |
| Trypsin-EDTA | Gibco | Cat#25200072 |
| Anti-F4/80 MicroBeadsUltraPure | Miltenyibiotec | Cat#130-110-443 |
| LS Columns | Miltenyibiotec | Cat#130-042-401 |
| Click-iT® EdU Flow Cytometry Assay Kits | Invitrogen | Cat#C10418 |
| BD Fixation/Permeabilization Kit | BD Biosciences | Cat#554714 |
| PinpoRNA RNA in-situ hybridization kit | GD Pinpoease Biotech Co | Cat # PIF2000 |
| Fatty Acid Oxidation (FAO) Colorimetric Assay Kit | Elabscience | Cat #E-BC-K784-M |
| Oleic Acid | Sigma-Aldrich | Cat#01008 |
| BIO1211 | TargetMol | Cat#T21868 |
| FFA free-BSA | Sigma-Aldrich | Cat#9048-46-8 |
| TNFα | Peprotech | Cat#315-01 A |
| CFSE | Invitrogen | Cat#65-0850-84 |
| Lipofectamine RNAiMAX | Thermo-Fisher Scientific | Cat#13778-150 |
| Experimental models: Organisms/strains | | |
| Mouse: C57BL6/J | Slac Laboratory Animal Inc | n/a |
| Mouse: B6. Cg-Cav1tm1Mls/J | The Jackson Laboratory | Cat#007083 |
| Mouse: *Trem2* knockout | Cyagen Biosciences | n/a |
| Mouse: B6/JGpt-H11em1Cin (CAG-LoxP-ZsGreen-Stop-LoxP-tdTomato)/Gpt | Gempharmatech | Cat#T006163 |
| Mouse: B6.FVB-Tg (Adipoq-cre)1Evdr/J | The Jackson Laboratory | Cat#028020 |
| Mouse:C57BL/6Smoc-*Cav1^em1(flox)Smoc^* | SHANGHAI MODEL ORGANISM | Cat#NM-CKO-2116185 |
| Mouse: B6.129P2-Lyz2tm1(cre)Ifo/J | The Jackson Laboratory | Cat#004781 |
| Oligonucleotides |  |  |
| Si-Cav-1 | Ribobio |  |
| Software and Algorithms |  |  |
| Prism 8 | GraphPad Software, Inc. | n/a |

**Table S2.** Primers sequence

| Gene | Primer sequence |
| --- | --- |
| *Col6a1*  *Itga1*  *Itgb5*  *Itgax*  *Mcam*  *Vcam1*  *Cd9*  *Cdh5*  *Cadm1*  *Pcdh1*  *Cav-1*  *Fabp4*  *Fabp5*  *Cd36*  *Cavin2*  *Cidec*  *Plin1*  *Plin4*  *Cd209d*  *Wfdc17*  *Pparg*  *Dgat2*  *Cyp2e1*  *Dcn*  *Sele*  *Ces1d*  *Mgll*  *Tnfa*  *Leptin*  *Adiponectin*  *Ccl5*  *Nos2*  *Acadm*  *Acads*  *Cact*  *Cpt1a*  *Cpt2*  *Hadha* | TCTTTTGAGTGCCAGGCTGC  CCTTCTCTCCCGGAAGACCT  TTTCCTGAGGACAGGAGACG  TTTCCTGAGGACAGGAGACG  CGCCTCGCAGGGCTCAA  TGGACCGTGGATTGCCAAAG  TCATCACTGATGGGAGAAAACA  CCCCAATTGCATAACGAATGAT  CAAGAGCTCAGGGAGGTTGC  CAAGAGCTCAGGGAGGTTGC  GCAAAGGACACTGGAAAAGAG  TCAAAGGGATACACATTAGGGAC  CCCTCATGATGCTGGTTGGT  AGGAACCCGAAGAACAATCCC  CACGGACAAGGCACCCG  GTGAGCCTCTGCATCCTTCG  TGCAGACCCAGCGCTATCTA  TTACCATCACAGGCTGGGG  GAGCACCCCGAGAATGAGCC  GGCGACGTCGGGCAAA  TTCTCTTAAATCACAGCCCAGG  TGTAGATGTTGCCCTGTTCC  AAGGTGAAGAGCATCATAACCCT  TCACGCCTTTCATAACACATTCC  GGTCAAAACCGAGAGCACAG  AGGTGCAGACCGTCTCAGT  GGATGGTTTCCTAGCCTTTCA  CGTGGCCCGGTTCTAATTCA  AGACTGGAGAACAACCACGC  AGGGATCTCACTTTCTTCCTGG  GTGAGGGGGAGGTCCAACA  CCGTGCTCACTGCCACAT  ACAGACACAGAGGGAGAGG  ACAGACACAGAGGGAGAGG  AAACAGCAACAGACCCCTC  AACTTCCCATGTCCTTGTCTC  GGATGGGTCTCTCAGACATGC  CCAATAGCGTGTAAAGCTGGG  TGGCTTTGATCACTGTGGGG  CCAGGTTTTTCAAATTCTTTGGGG  TCGCTGATGCACTGCCTATG  GAGAGGTCCACAGAGCTGATT  GAACACGCCCAAGAAAGGTGG  GGTGGTCAGCAGGTTGTGTG  CAGGAGTACAAGAACAAGGGGAT  TTTGGATGCGGGCCTCATTA  TCCGACTTCAATGGACTGAAC  TGAGGGATCGCAGTTATGTTG  GCAAAGCTGTCCAGTGTGAA  TCGCAGGAGAACTCACAACT  AGGACTTGATGGCTGATGTG  GGTCTCCTATTACTGCCTTGG  GGAACAAGTCGGAGGGTTCT  CCGGGATGATTCCATGAGCA  CCCTCCAGAAAAGACACCATG  TTGAGATCCATGCCGTTGG  GTGCCTATCCAGAAAGTCCAG GACCTGTTGATAGACTGCCAG  TGTTCCTCTTAATCCTGCCCA CCAACCTGCACAAGTTCCCTT  GCCCACGTCAAGGAGTATTT CTTGAACCCACTTCTTCTCTGG  TCCTGGACATTACGACCCCT  GATCACTCAGCTGAAAATCGAC  AGGGTTTAGTTTTGAGTTGACGG  CCCCGCTTTTGTCATATTCCG  GACTGGCGACGGTTACACA  GGCAAAGTCACGGCATGTC  GACGAGCCGAAACCCATCAG  AGTCGGACCTTGACCGTGT  CTCCGCCTGAGCCATGAAG  CACCAGTGATGATGCCATTCT  CAGCACAGCATCGTACCCA  TCCCAATGCCGTTCTCAAAAT  GAGCTTTCGTCCTCTTCTGCT  CTGAAGGCACTCCGAGACT |
